# Reduced Cx43 S-nitrosylation protects against cardiac stress–induced arrhythmias and myocardial injury in Duchenne muscular dystrophy

**DOI:** 10.1093/cvr/cvag170

**Published:** 2026-08-10

**Authors:** Manuel F Muñoz, Pablo S Gaete, Jonathan J Quan, Thao T Nguyen, Janet Nuno, Adrian Sheehy, Priscila A Araujo, Pia C Burboa, Mauricio A Lillo, Jorge E Contreras

**Affiliations:** Department of Physiology and Membrane Biology, University of California, One Shields Avenue, Davis, CA 95616, USA; Department of Physiology and Membrane Biology, University of California, One Shields Avenue, Davis, CA 95616, USA; Department of Physiology and Membrane Biology, University of California, One Shields Avenue, Davis, CA 95616, USA; Department of Physiology and Membrane Biology, University of California, One Shields Avenue, Davis, CA 95616, USA; Department of Physiology and Membrane Biology, University of California, One Shields Avenue, Davis, CA 95616, USA; Department of Physiology and Membrane Biology, University of California, One Shields Avenue, Davis, CA 95616, USA; Department of Physiology and Membrane Biology, University of California, One Shields Avenue, Davis, CA 95616, USA; Department of Pharmacology, Physiology and Neuroscience, Rutgers University, New Jersey Medical School, Newark, USA; Department of Pharmacology, Physiology and Neuroscience, Rutgers University, New Jersey Medical School, Newark, USA; Department of Physiology and Membrane Biology, University of California, One Shields Avenue, Davis, CA 95616, USA

**Keywords:** Connexins, S-nitrosylation, Myocardial injury, Arrhythmias, Cardiomyopathies

## Abstract

**Aims:**

In Duchenne muscular dystrophy (DMD), connexin-43 (Cx43) delocalizes from intercalated discs to the lateral membrane of cardiomyocytes, where it forms undocked hemichannels. β-adrenergic stress induces lethal arrhythmias in DMD^mdx^ mice that correlate with increased Cx43 S-nitrosylation and excessive hemichannel opening. Here, we aimed to determine whether S-nitrosylation of Cx43 at cysteine 271 directly contributes to β-adrenergic stress–induced arrhythmias and myocardial injury in DMD.

**Methods and results:**

To define the role of Cx43 S-nitrosylation in DMD cardiomyopathy, we generated DMD^mdx^ knock-in mice carrying a cysteine-to-serine substitution at residue 271, a critical S-nitrosylation site involved in NO-dependent Cx43 hemichannel activation (DMD^mdx^:C271S^+/−^). Following β-adrenergic stimulation, DMD^mdx^ mice exhibited a higher incidence of arrhythmogenic events and increased triggered activity in isolated cardiomyocytes compared with DMD^mdx^:C271S^+/−^ mice, which more closely resembled wild-type mice. Optical mapping of isolated hearts showed that DMD^mdx^ mice developed abnormal Ca^2+^ transients, prolonged action potentials, and episodes of conduction block. These abnormalities were normalized in DMD^mdx^:C271S^+/−^ mice and attenuated by treatment with the Cx43 hemichannel inhibitor Gap19. Moreover, β-adrenergic stress evoked severe myocardial injury and bradycardia in DMD^mdx^ mice, both of which were significantly attenuated in DMD^mdx^:C271S^+/−^ mice. Notably, Gap19-treated DMD^mdx^ mice were protected against myocardial injury, further supporting a direct role for Cx43 hemichannels in DMD-associated cardiac damage.

**Conclusion:**

These findings reveal, for the first time, a mechanistic link between Cx43 hemichannel activity, arrhythmogenesis, and myocardial injury in DMD. We conclude that S-nitrosylation of Cx43 is a fundamental NO-mediated mechanism driving β-adrenergic stress–induced arrhythmias and myocardial injury in DMD^mdx^ mice through pathological opening of Cx43 hemichannels.

**Time of primary review: 35 days**

**See the editorial comment for this article ‘Muting lateralized Cx43 hemichannels to rescue the stressed Duchenne heart’, by M. Badaoui, https://doi.org/10.1093/cvr/cvag194.**

Translational perspective DMD cardiomyopathy remains a leading cause of mortality, yet therapies that directly prevent stress–induced arrhythmias and myocardial injury remain limited. Our findings identify Cx43 S-nitrosylation and hemichannel opening as actionable mechanisms that drive β-adrenergic stress–induced cardiac dysfunction in dystrophic hearts. By linking a specific post-translational modification of Cx43 to arrhythmogenic activity and myocardial damage, this work provides a mechanistic framework for therapeutic intervention. Pharmacological inhibition of Cx43 hemichannels may therefore offer a targeted, disease-relevant strategy to reduce arrhythmogenic risk, preserve cardiac function, and improve clinical outcomes in patients with DMD.

## 1. Introduction

Duchenne muscular dystrophy (DMD) is a severe and progressive muscle-wasting disease that primarily affects males, occurring in approximately 1 in 5000 male newborns.^1,2^ It is characterized by an X-linked mutation leading to the loss of function in the dystrophin gene, which encodes a crucial protein responsible for maintaining membrane stability in muscle cells.^2^ DMD patients typically present with muscle weakness, respiratory failure, and cardiac dysfunction, with cardiomyopathies, particularly dilated cardiomyopathy,^3^ being the leading cause of mortality.^1^ Among the primary clinical heart complications observed in these patients are acute myocardial injury,^4,5^ elevated plasma levels of cardiac troponin-I (cTnI),^6^ and significant sympathetic activity.^7^ Despite extensive research, the molecular mechanisms underlying DMD cardiomyopathy remain incompletely understood. Current therapeutic approaches, such as gene therapy using adeno-associated virus-based methods to restore dystrophin,^8^ losartan treatment to antagonize angiotensin II type I receptors,^9–11^ and β-receptor blocker therapy^12^ have only partially reduced the cardiac dysfunction and associated mortality in these patients. This represents a critical unsolved challenge that must be addressed to improve the lives of DMD patients.

In the heart, connexin-43 (Cx43) is the predominant connexin isoform expressed in the ventricular myocardium.^13,14^ Intriguingly, Cx43 is extensively remodelled in DMD cardiomyopathy, exhibiting abnormal lateral distribution in cardiomyocytes.^15–19^ Our laboratory has shown that hyperactive remodelled Cx43 hemichannels critically contribute to arrhythmogenicity in a dystrophic mouse model (DMD^mdx^).^18–21^ We reported that DMD^mdx^ mice with lower levels of Cx43 proteins (DMD^mdx^ mice: Cx43^+/−^)^20^ or the blockade of Cx43 hemichannels using connexin mimetic peptides displayed a low incidence of arrhythmias and sudden cardiac death following isoproterenol challenge.^22^ Cx43 hemichannel blockade prevented cardiac stress-elicited membrane depolarization and triggered activity (TA) in DMD^mdx^ cardiomyocytes, both key drivers of arrhythmogenesis.^19^ Notably, Cx43 hemichannels have also been implicated in infarct size and cardiomyocyte viability following ischaemia/reperfusion injury in both *in vivo* and *in vitro* models.^23–25^ DMD patients and mouse models exhibit myocardial injury as a hallmark of cardiomyopathy throughout life.^6,26–29^ However, the role of Cx43 hemichannels in this process remains unexplored, despite multiple reports of myocardial injury in DMD patients and the lack of clarity regarding its underlying mechanisms.^4–7^

Previously, we and others reported that stimulation with isoproterenol promotes hyper-nitrosylation of Cx43 proteins in the hearts of DMD^mdx^ mice,^19,30^ which was prevented by treating the animals with the nitric oxide (NO) synthase inhibitor, N(ω)-nitro-L-arginine methyl ester (L-NAME).^19^ L-NAME also prevented isoproterenol-induced arrhythmias and TAs in DMD^mdx^ mice. However, this treatment equally affects S-nitrosylation levels of other key proteins implicated in sarcoplasmic reticulum Ca^2+^ leaks and ventricular arrhythmias, including ryanodine receptor type 2 (RyR2),^31^ Ca^2+^/calmodulin-dependent protein kinase II (CaMKII),^32^ voltage-gated Na^+^ channels,^33^ and other proteins associated with cardiac dysfunction and Ca^2+^ handling.^34^ Thus, the precise role of Cx43 S-nitrosylation in DMD-related cardiomyopathy remains unresolved. Electrophysiological studies using *in vitro* heterologous expression systems revealed that NO induces S-nitrosylation of Cx43 at cysteine 271 (C271), which is critical for promoting the opening of Cx43 hemichannels.^19^ Therefore, we hypothesize that S-nitrosylation of lateralized Cx43 hemichannels at C271 is critical for promoting excessive Ca^2+^ influx, membrane depolarization, and TA after β-adrenergic stimulation in DMD^mdx^ cardiomyocytes. To test this hypothesis, we developed a novel DMD^mdx^ knock-in mouse model with reduced levels of S-nitrosylated Cx43 (DMD^mdx^:C271S^+/−^). Our results show that specifically impairing Cx43 S-nitrosylation in DMD^mdx^ mice is sufficient to restore cardiac excitability and prevent lethal arrhythmias. Furthermore, we provide the first evidence that Cx43 hemichannels contribute to cardiac stress–induced rhythm dysfunction and myocardial injury. These results unequivocally establish S-nitrosylated Cx43 hemichannels as key mediators of arrhythmic events and myocardial cell viability loss.

## 2. Methods

### 2.1 Animals

DMD^mdx^ mice (4–6 months old; males and females) were obtained from Jackson Laboratory. Mice were heparinized (5000 U/kg) and then anesthetized with 4–5% isoflurane (Isospire, ANADA #200-129) for induction before surgery, followed by 1–5% for maintenance. We recently published that the S-nitrosylation of Cx43 occurs specifically at cysteine 271 (C271) at the C-terminus of Cx43.^19^ Consistent with this finding, we generated a global knock-in mouse line using CRISPR-Cas9 genome editing technology, in which the site of S-nitrosylation of Cx43 is substituted by serine (C271S). After generating stable colonies of heterozygous C271S^+/−^ and homozygous C271S^+/+^ mice, normal growth, breeding, and survival were observed. Finally, DMD^mdx^ mice were crossed to C271S colonies, obtaining DMD^mdx^:C271S^+/−^ mice. Genotyping was performed using automated Transnetyx. Mice were provided with food and water *ad libitum*. All experiments involving mice were approved by the Institutional Animal Care and Use Committee (IACUC) of the School of Medicine at the University of California, Davis, and performed in accordance with the National Institutes of Health guidelines.

### 2.2 Immunofluorescence in cryosections of mouse heart ventricles

Mouse ventricles were quick-frozen in liquid nitrogen and stored at −80°C; 6 µm sections of mouse ventricles were obtained from cryosectioning. Samples were thawed at room temperature (RT) and washed three times with 1 × phosphate buffered saline (PBS). Heart samples were then fixed with 4% paraformaldehyde (PFA) for 10 min and washed three times with 1 × PBS. Fixed hearts were permeabilized with 0.5% Triton X-100 in PBS for 30 min, followed by three washes with PBSt (1 × PBS containing 0.1% Tween-20). Subsequently, samples were incubated with blocking buffer, 10% normal goat serum, for 1 h at RT (#005-000-121, Jackson ImmunoResearch). Sections were incubated with Cx43 (1:1000 dilution, anti-rabbit, #C6219, Sigma-Aldrich) and N-cadherin (1:100 dilution, anti-mouse, #33-3900, Invitrogen) antibodies in blocking buffer at 4°C overnight. Heart sections were washed with PBSt and then incubated with goat Alexa Fluor secondary antibodies diluted 1:200: Alexa Fluor 555 goat anti-mouse IgG (#A21424, Invitrogen) and Alexa Fluor 488 goat anti-rabbit IgG (#A11008, Invitrogen). Sections were washed with PBSt and incubated with Wheat Germ Agglutinin (WGA) conjugated to Alexa Fluor 350 (40 µg/mL, #W11263) for 1 h to mark the cell membrane of cardiomyocytes. Lastly, coverslips were mounted on heart sections using ProLong™ Gold antifade reagent (#P36930, Invitrogen). Images were taken on a Zeiss LSM880 Airyscan confocal microscope and analysed using the software ImageJ. For each experiment, the percentage of Cx43 outside the intercalated discs (% Cx43 outside ID) was calculated as the proportion of the total Cx43 signal area that does not colocalize with N-cadherin, an intercalated disc marker, relative to the total Cx43 signal area.

### 2.3 Electrocardiogram telemetry

Mice were anesthetized with 4% isoflurane for induction, followed by 2% for maintenance. Electrocardiograms (ECGs) were recorded in lead II using a subcutaneously implanted wireless radio telemeter (ETA-F10 from DSI). Buprenorphine (0.05–0.1 mg/kg) was injected subcutaneously after the surgery and then as needed if signs of pain were present. Mice recovered for 3 days, and on the 4th day post-electrode implantation, they were first weighed and then separated into single cages placed on telemetry receivers. Mice were treated with isoproterenol (5 mg/kg i.p.; no more than 0.25 mL; #1351005, USP), and ECGs were continuously recorded and evaluated over a 24-hour post-injection period. A 1-hour baseline reading was taken to monitor activity and obtain ECG recordings. All cardiac parameters were measured using the ER400 energizer/receiver, and data were collected using Ponemah software. The total number of arrhythmias included single premature ventricular contractions (PVCs), double PVCs, and three or more PVCs (non-sustained ventricular tachycardia, NSVT).

### 2.4 Ca^2+^ transients and Δ*V_m_* changes in optical mapping in the intact mouse heart

The mice were anaesthetized using isoflurane and euthanized by cervical dislocation. The hearts were rapidly isolated and then Langendorff-perfused through the aorta with a warmed and oxygenated KH solution (in mM: 119 NaCl, 25 NaHCO_3_, 4 KCl, 1.2 KH_2_PO_4_, 1 MgCl_2_, 1.3 CaCl_2_·2H_2_O, 10 D-glucose) at a flow rate of 3 mL/min, maintaining a temperature of 37°C with a gas mixture of 95% O_2_ and 5% CO_2_. Once the heart was cannulated, it was placed in the optical mapping setup (MappingLab, UK). Optical mapping recordings were captured using two high-speed sCMOS cameras (Prime BSI Express, Teledyne Photometrics) with high quantum efficiency. For imaging, heart preparations were excited at 530 ± 20 nm using a light-emitting diode epi-illumination. The emission light passing through a premium long-pass filter (FELH550) was split using a dichroic mirror (Thorlabs; DMLP638). The emission light was then passed through a 700 nm long-pass filter (FELH700) and a 585 nm band-pass filter (Thorlabs; FBH585) for Δ*V_m_* and Ca^2+^ imaging, respectively. To prevent motion artefacts, hearts were mechanically uncoupled using 4 µM blebbistatin (#TRC-B592500, TRC) and 0.008% Pluronic acid F-127 (#540025, Millipore) following a previously described protocol.^35^ Simultaneous recording of Δ*V_m_* and Ca^2+^ transients was performed after loading the hearts with RH237 (N-(4-Sulfobutyl)-4-(6-(4-(Dibutylamino)phenyl)hexatrienyl)Pyridinium, Inner Salt, #S1109, ThermoFisher) and Rhod-2AM (#R1244, ThermoFisher), respectively. Pharmacological agonists were introduced directly into the perfusion after baseline recordings were obtained, and recordings were evaluated immediately after 10 Hz stimulation in the presence of 100 nM isoproterenol, and 2 min post-stimulation, representing the recovery time. During stimulation, electrodes were strategically positioned in the ventricular region of the heart, and hearts were stimulated at 10 Hz at a constant current of 1 mA for 1 min. Ca^2+^ transient amplitudes and action potential durations (APDs) were calculated as the average of six independent regions of interest (ROIs) in the ventricular area of the mouse heart. The coefficient of variation (COV) of diastolic intervals was measured as the standard deviation divided by the mean diastolic interval. The diastolic intervals were obtained from diastolic time intervals among action potentials (APs), by measuring the time from APD_90_ to the activation time of the subsequent AP,^36,37^ calculated as the average of six consecutive beats in six independent ROI in the ventricular area of the mouse heart. Fluorescence images were captured at a sample rate of 1200 fps, with a region size of 150 × 150 pixels and an exposure time of 0.7 ms. Optical mapping data were acquired in real-time using an 8-channel TTL analogue-to-digital converter with OMapRecord 5.0 software and processed with OMapScope5.9.4 (Mapping Lab). Assessment of Δ*V_m_* and Ca^2+^ dynamics during β-adrenergic receptor overstimulation was conducted following previously established protocols, with specific modifications. The composition of the KH solution remained unchanged, except for an increase in CaCl_2_ concentration from 1.3 mM to 1.8 mM. Isoproterenol was introduced as a β-adrenergic receptor agonist at a final concentration of 4 μM to induce receptor overstimulation.

### 2.5 Measurement of plasma cTnI

Blood was collected from the tail vein of isoflurane-anesthetized mice before and 4 h after the isoproterenol injection. The blood volume collected from the tail vein was limited to 80 to 120 µL per collection, in accordance with UC Davis IACUC-approved guidelines. The blood samples were stored in EDTA-containing tubes and kept on ice. Following centrifugation at 4000 RPM for 20 min, plasma samples were stored at −80°C until ELISA analysis. Serum cTnI levels were measured using the high-sensitivity mouse cTnI ELISA Kit (#CTNI-1-HSP, Life Diagnostics, Inc.) on the multimode plate reader, VICTOR Nivo (PerkinElmer Life).

### 2.6 2,3,5-Triphenyltetrazolium chloride staining

2,3,5-Triphenyltetrazolium chloride (TTC) staining was performed to evaluate myocardial injury.^38^ After heart isolation, hearts were kept at −20°C for 1 h. Using a razor blade, the hearts were cut transversely into 2-mm-thick slices. The slices were then incubated in 1% TTC in 1× PBS for 1 h at 37°C, followed by incubation in 4% PFA for 30 min at RT. Finally, the slices were transferred to 1× PBS. Pictures were taken using a stereoscopic microscope coupled with a camera at 20× magnification.

### 2.7 Retro-orbital injection of Gap19

Gap19 (10 mg/kg) was administered *in vivo* under anaesthesia via retro-orbital injection 30 min before isoproterenol administration (5 mg/kg, via intraperitoneal injection, IP). The mouse weight was between 25 and 30 g, and the plasma volume was approximately 1.5 mL. Therefore, the estimated plasmatic concentration of Gap19 is approximately 167 µM, following the injection of a volume of ∼100 µL. Saline solution (0.9% NaCl) was utilized as a control vehicle and administered via retro-orbital injection.^22^ For optical mapping experiments, Gap19 (10 mg/kg) was administered *in vivo* via retro-orbital injection 1–2 h before heart isolation to ensure myocardial delivery before *ex vivo* recordings. Hearts were then excised and used for optical mapping.

### 2.8 Molecular biology

cDNA for human Cx43 was synthesized and subcloned into a pGEM-HA vector. The single mutation C271S in Cx43 was introduced using the QuickChange II Site-Directed Mutagenesis Kit (Agilent Technologies) and confirmed by Sanger sequencing. cDNAs were transcribed *in vitro* to cRNAs using the HiScribe^®^ T7 Arca mRNA Kit (New England BioLabs). A Cx38 antisense oligonucleotide (Sequence: 5ʹ GCT TTA GTA ATT CCC ATC CTG CCA TGT TTC 3ʹ^39^) was synthesized by Integrated DNA Technologies (Coralville, IA).

### 2.9 Expression of Cx43 in Xenopus oocytes

Oocytes from female *Xenopus laevis* (Xenopus 1 Corp, Dexter, MI) were collected and digested as we described previously^40,41^ in strict adherence to the protocol approved by the IACUC at the University of California Davis and conforming to the National Institutes of Health Guide for the Care and Use of Laboratory Animals. Defolliculated oocytes (stage IV-V) were individually microinjected with 25 ng of cRNAs for human Cx43 wild-type (WT), human Cx43 C271S, or a combination of Cx43 WT plus Cx43 C271S (ratio 1:1). Oocytes were also injected with an antisense oligonucleotide (AS) to prevent the expression of endogenous Xenopus Cx38, as previously described by Ebihara.^39^ Non-injected oocytes or oocytes injected with AS alone were used for control experiments. After microinjection, oocytes were stored for 2 days at 16°C in sterile plates containing ND96 solution (composition in mM: 96 NaCl, 2 KCl, 1 MgCl_2_, 1.8 CaCl_2_, 5 HEPES, adjusted to pH 7.4) supplemented with streptomycin (50 µg/mL) plus penicillin (50 Units/mL).

### 2.10 Two-electrode voltage clamp

The two-electrode voltage clamp (TEVC) technique was performed as previously described by us.^41^ Briefly, two pulled borosilicate glass micropipettes were filled with 3 M KCl, resulting in resistances ranging from 0.2 to 2 MΩ. The electrical signal was amplified using an Oocyte Clamp Amplifier (OC-725C, Warner Instrument Corp., USA) and digitized by a data acquisition system (Digidata 1440A, Molecular Devices, USA). Data were sampled at 2 kHz, and analysed using pClamp 10 software (Molecular Devices, USA). Experiments were conducted at RT (20–22°C). The recording solution contained (in mM) 117 Tetraethylammonium chloride (TEACl), 0.2 CaCl_2_, and 5 HEPES, with the pH adjusted to 7.40. The current-voltage relationship was obtained by analysing the magnitude of activation currents evoked by depolarizing pulses (from −80 to +80 mV; holding potential: −80 mV; duration of depolarization pulse: 2 s). After recording ionic currents under control conditions, oocytes were incubated with 100 µM diethylamine NONOate (DEA NONOate), a NO donor used to increase ionic currents at potentials near the membrane potential in TEVC experiments, for 15 min, and ionic currents were recorded again. To confirm that currents were mediated by Cx43, we incubated the oocytes with 200 µM lanthanum chloride (LaCl_3_) in the presence of DEA NONOate at the end of each experiment. Only those experiments with La^3+^-sensitive currents were considered for the analysis of the Cx43 WT group.

### 2.11 Biotin switch for S-nitrosylated proteins

To evaluate S-nitrosylation levels of Cx43 proteins in knock-in mice, we isolated S-nitrosylated Cx43 proteins from ventricular tissue obtained from WT, DMD^mdx^, DMD^mdx^:C271S^+/−^, C271S^+/−^, and C271S^+/+^ mice. Firstly, heart samples were homogenized and solubilized according to the biotin-switch protocol using HEN buffer (250 mM HEPES, 0.1 mM EDTA, 0.1 mM neocuproine, and protease inhibitor cocktail, pH = 7.7). Biotin-switch protocol^42^ was performed on 200 µg of protein samples from WT, DMD^mdx^, DMD^mdx^:C271S^+/−^, C271S^+/−^, and C271S^+/+^ mice. Briefly, heart samples were treated with S-methyl methanethiosulfonate (#208795, Sigma-Aldrich) at 50°C for 1 h to block cysteine-free thiols. Subsequently, proteins were precipitated by incubating them in acetone at −20°C for 24 h. Following precipitation, protein pellets were washed and resolubilized in HEN buffer before biotinylation. Heart samples underwent incubation with Biotin-HPDP (#16459, Cayman Chemicals) and sodium L-ascorbate (#11140, Sigma-Aldrich) to convert S-nitrosylated cysteine residues into free cysteine. Proteins were precipitated again to remove any excess of Biotin-HPDP and solubilized to run western blots. After solubilization, the heart samples were incubated with Streptavidin Agarose Resin (#20349, ThermoScientific) and then centrifuged at 24 000 RPM for 20 min at 4°C to pull down the respective biotin-tagged proteins. The proteins were subsequently separated via electrophoresis using 12% SDS-PAGE gels and transferred onto a PVDF membrane (Trans-Blot Turbo, Bio-Rad). S-nitrosylated Cx43 proteins were detected using a mouse anti-Cx43 antibody (#C8093, Sigma-Aldrich, dilution: 1:8000), and GAPDH was detected using a rabbit anti-GAPDH antibody as a loading control (#ab181603, Abcam, dilution: 1:1000). ImageJ software was used to analyse the intensity of the signal.

### 2.12 Measurement of arterial blood pressure

To measure arterial blood pressure, mice were anesthetized using isoflurane at a concentration of 1.5%, ensuring deep anaesthesia was achieved, as indicated by the absence of reflexes. The anesthetized mice were placed on a heating pad in a supine position, and the surgical field was prepared with sterile drapes. A midline incision was performed in the neck to expose the carotid artery. A sterile catheter connected to a pressure transducer (ADInstruments, Australia) was inserted into the artery. The catheter was secured and connected to the pressure transducer, and the system was flushed with sterile or heparinized saline to remove any air bubbles. Arterial blood pressure was recorded using LabTutor (version 2012), the pressure transducer was calibrated, and proper zeroing was ensured. Throughout the data collection process, both the mouse and the catheter were continuously monitored to ensure stable measurements. After data collection was completed, the catheter was carefully removed, the incision was closed with sutures or surgical glue, and sterile eye ointment was applied. Pressure changes were assessed 30 min post-surgery by calculating an average of four independent measurements taken every 10 min.

### 2.13 Isolation of ventricular myocytes

Cardiomyocytes were isolated enzymatically from adult mouse hearts using a Langendorff-based retrograde perfusion approach. Mice were injected with heparin (5000 U/kg) and anesthetized with a mixture of ketamine/xylazine. After heart excision, the aorta was cannulated and perfused with a Tyrode solution (composition in mM: NaCl 130, KCl 5, NaH_2_PO_4_ 0.33, MgSO_4_ 0.5, glucose 10, and HEPES 10, adjusted to pH 7.4) containing 50 µM CaCl_2_ and collagenase type II (1 mg/mL; Worthington). After enzymatic digestion, hearts were removed from the perfusion system, and the ventricle was dissociated in a petri dish using fine forceps. The resulting cell suspension was filtered through nylon mesh, and extracellular Ca^2+^ was reintroduced gradually to a final concentration of 1.0 mM. Isolated cardiomyocytes were maintained at RT and used within 6 h of isolation.

### 2.14 Patch-clamp electrophysiology

Membrane potential and ionic currents were recorded from isolated ventricular cardiomyocytes using the whole-cell patch-clamp technique. For voltage clamp experiments, pipettes (2–4 MΩ) were filled with a Cs^+^-based internal solution containing (in mM): 110 Cs-methanesulfonate, 20 TEACl, 1 MgCl_2_, 10 HEPES, 10 EGTA, and 5 MgATP. Cells were superfused with an external solution containing (in mM): 140 NMDG, 5 CsCl, 2 CaCl_2_, 1 MgCl_2_, 10 glucose, and 10 HEPES, pH 7.4. Voltage pulse consisted of a holding potential of −80 mV, followed by a 150 ms step to −50 mV and then 300 ms depolarizing steps up to +40 mV. Ca^2+^-currents were normalized to membrane capacitance and confirmed by nifedipine sensitivity.

For current-clamp experiments, pipettes (1.5–3 MΩ) were filled with internal solution containing (in mM) 110 K-aspartate, 30 KCl, 5 NaCl, 0.1 EGTA, 5 Mg-ATP, 10 HEPES, and 5 Na_2_-creatine phosphate, pH 7.3, ∼290 mOsm. Cells were superfused with Tyrode solution containing (in mM) 136 NaCl, 5.4 KCl, 0.33 NaH_2_PO_4_, 1 CaCl_2_, 1 MgCl_2_, 10 glucose, and 10 HEPES, adjusted to pH 7.4. APs were elicited by 2-ms depolarizing current pulses of 2 nA delivered at 1 Hz. Experiments were performed at 37°C with a flow of 2 mL/min.

### 2.15 Chemicals

Isoproterenol Hydrochloride was obtained from USP (#1351005). (S)-(−)-Blebbistatin was purchased from LGC standard (#TRC-B592500). LaCl_3_ (#203521) and diethylamine NONOate sodium salt hydrate (DEA NONOate) (#D184) were obtained from Sigma-Aldrich. Heparin was obtained from Fresenius Kabi (NDC #63323-047-01). The connexin 43 hemichannel blocker, Gap19 (#5353), was obtained from Tocris, and the scrambled Gap19 peptide (Sequence: IKFKKEIKQ) was synthesized in LifeTein.

### 2.16 Statistical analysis

The data are presented as mean ± standard error of the mean. Group comparisons were performed using an unpaired *t*-test, one-way ANOVA, or two-way ANOVA, as appropriate, followed by Tukey’s *post-hoc* test for multiple comparisons when applicable. A significance level of *P* < 0.05 was used to determine statistical significance. *P* values from statistical analyses are indicated in each figure. Statistical analyses were performed using OriginPro 9.85.

## 3. Results

### 3.1 Generation and characterization of C271S^+/−^ and DMD^mdx^:C271S^+/−^ mouse lines

We previously demonstrated that NO induces S-nitrosylation of Cx43 at residue C271 in heterologous expression systems.^19^ To evaluate whether substitution of C271 with serine alters Cx43 hemichannel opening, we measured ionic currents from Cx43 WT and mutant C271S hemichannels expressed in Xenopus oocytes using two-electrode voltage clamp.^43^ WT Cx43-expressing oocytes displayed increased ionic currents when exposed to the NO donor 100 µM DEA NONOate, even at potentials close to cardiomyocyte membrane potentials (−70 mV)^18^ (see Supplementary material online, *Figure S1*). In contrast, oocytes expressing either the Cx43 mutant (Cx43^C271S^) or co-expressing both the WT and C271S mutant (Cx43^WT/C271S^) displayed no significant ionic currents under the same conditions. This indicates that the C271S mutation abolishes NO-induced activation of Cx43 hemichannels.

To assess the *in vivo* effects of S-nitrosylation in Cx43 hemichannels at cysteine 271, we developed a mouse model in which this site was replaced with serine (C271S) using CRISPR-Cas9 genome editing (see Supplementary material online, *Figure S2A*). The generation of the C271S mutation produced WT (C271S^−/−^), heterozygotes (C271S^+/−^), and homozygotes C271S (C271S^+/+^) mice. Supplementary material online, *Figure S2B* shows a representative biotin-switch assay that confirms reduced or zero levels of S-nitrosylation in C271S^+/−^ and C271S^+/+^ mice, respectively. Importantly, C271S^+/−^ and C271S^+/+^ mice showed no difference in animal weight, heart weight, and heart/animal weight ratio with respect to WT mice (see Supplementary material online, *Figure S2C*, *S2D*, and *S2E*). However, C271S^+/+^ mice displayed elevated blood pressure (138.5 ± 3.14 mmHg) in comparison to WT (101.5 ± 0.92 mmHg) and C271S^+/−^ mice (105.6 ± 3.74 mmHg) (see Supplementary material online, *Figure S2F*), indicating off-target effects that might eventually affect cardiac function. This precludes the use of C271S^+/+^ mice for rigorously establishing the relationship between S-nitrosylated Cx43 hemichannels and DMD^mdx^ cardiomyopathy. Based on our electrophysiological findings, the C271S mutation serves as a dominant negative for NO-dependent Cx43 hemichannel opening (see Supplementary material online, *Figure S1*). Therefore, we generated a DMD^mdx^ knock-in mouse with reduced S-nitrosylation of Cx43 using the heterozygote C271S^+/−^ mice. This model is referred to as DMD^mdx^:C271S^+/−^ mice (see Supplementary material online, *Figure S3*). Importantly, we observed no weight differences among 4–6-month-old WT, DMD^mdx^, and DMD^mdx^:C271S^+/−^ mice (see Supplementary material online, *Figure S4*). Remarkably, DMD^mdx^:C271S^+/−^ mice did not develop the hypertrophy characteristic of DMD^mdx^, exhibiting heart weight and heart-to-body weight ratios comparable to those of WT and C271S^+/−^ mouse lines (see Supplementary material online, *Figure S4*), suggesting a protective effect against hypertrophy, a well-known characteristic observed in DMD^mdx^ mice.^44^

### 3.2 β-adrenergic stimulation evokes severe arrhythmias in DMD^mdx^ mice, which are prevented in DMD^mdx^:C271S^+/−^ mice

Arrhythmogenicity observed in DMD^mdx^ hearts upon β-adrenergic stimulation correlates with increased S-nitrosylation of Cx43, both of which are inhibited by L-NAME, a NOS blocker.^19^ However, the specific impact of reduced S-nitrosylation of Cx43 at cysteine 271 on arrhythmogenesis during isoproterenol-induced cardiac stress *in vivo* has not been explored. To investigate this, we performed ECG telemetry recordings to analyse arrhythmogenic events in WT, DMD^mdx^, and DMD^mdx^:C271S^+/−^ mice under control conditions and for 24 h post-isoproterenol injection (5 mg/kg). Representative ECG recordings at baseline, 1 h, and 24 h post-isoproterenol from WT, DMD^mdx^, and DMD^mdx^:C271S^+/−^ mice are shown in *Figure 1A*. *Figure 1B* shows the increased number of total arrhythmogenic events during the 24 h post-isoproterenol in DMD^mdx^ mice (212 ± 42.21 events) compared with WT mice (31.14 ± 5.81 events). In contrast, DMD^mdx^:C271S^+/−^ mice exhibited a significantly reduced number of arrhythmogenic events (92.4 ± 18.38 events) compared with DMD^mdx^ mice. The total arrhythmogenic events included single PVCs (*Figure 1C*, left panel), double PVCs (*Figure 1C*, middle panel), and NSVT (*Figure 1C*, right panel). Collectively, our data demonstrate that DMD^mdx^:C271S^+/−^ mice exhibit anti-arrhythmic protection against β-adrenergic-mediated cardiac stress observed in DMD^mdx^ mice.

**Figure 1:**
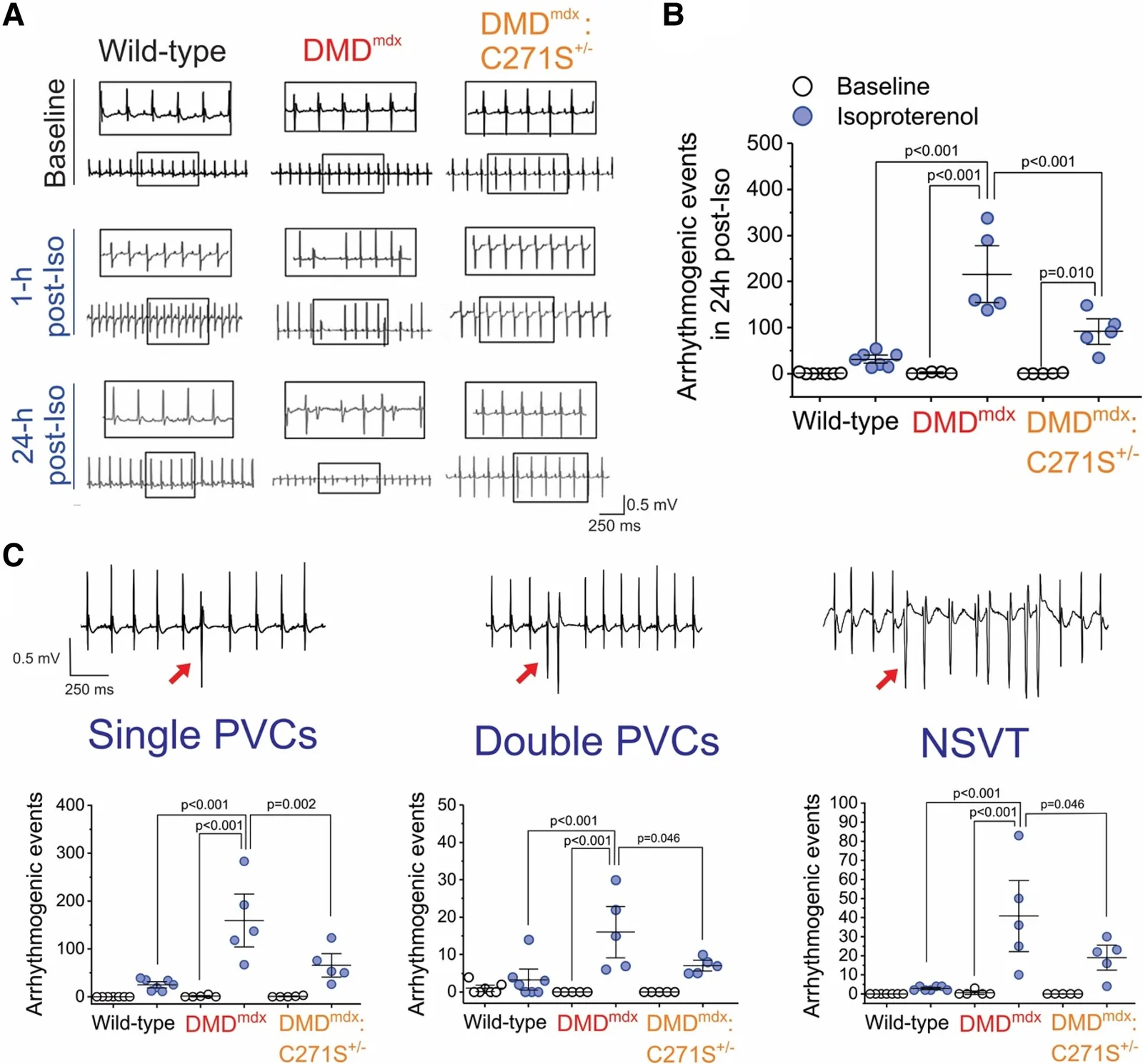
Reduced levels of S-nitrosylation of Cx43 hemichannels rescue the deadly DMD arrhythmogenicity induced by β-adrenergic stimulation-mediated cardiac stress. (*A*) Representative ECG recordings from WT, DMD^mdx^, and DMD^mdx^:C271S^+/−^ mice via telemetry implants. The top trace represents recordings obtained at baseline conditions. Traces in the middle and bottom indicate 1- and 24-h post-isoproterenol (5 mg/kg) challenge injected intraperitoneally, respectively. Scale bar = 250 ms. (*B*) Quantification of the total number of arrhythmogenic events before (baseline, white circles) and during the 24-h post-isoproterenol injection (isoproterenol, blue circles). (*C*) Quantification of the type of arrhythmogenic events induced by β-adrenergic stimulation: single PVCs (left), double PVCs (middle), and NSVT (three or more consecutive PVCs) (right). The type of arrhythmia can be graphically observed at the top of each graph, indicated by a red arrow. *n* = 5–7 mice per group. Each dot represents an independent animal. Group comparisons were made using a two-way ANOVA followed by Tukey’s *post-hoc* test. Data are presented as means ± SEM.

### 3.3 Remodelling of Cx43 is not prevented in hearts from DMD^mdx^:C271S^+/−^ mice

We have previously demonstrated that Cx43 remodelling is mediated by hypophosphorylation of serine residues 325, 328, and 330 and that it is a critical factor for the development of cardiac dysfunction and arrhythmogenicity in DMD^mdx^ mice.^18^ Therefore, we investigated whether S-nitrosylation of Cx43 at residue C271 also contributes to this remodelling in DMD^mdx^ mice. To evaluate Cx43 distribution in the ventricles, we performed immunostaining for Cx43 (green), the intercalated disk marker N-cadherin (red), and the cell membrane marker WGA (blue) in ventricular sections of 4–6-month-old WT, DMD^mdx^, DMD^mdx^:C271S^+/−^, and C271S^+/−^ mice. Representative confocal images (*Figure 2A*) show that Cx43 is predominantly localized to the lateral side of cardiomyocytes in DMD^mdx^ and DMD^mdx^:C271S^+/−^ hearts, whereas it remains primarily at the intercalated disc in WT and C271S^+/−^ hearts. Quantification of the percentage of Cx43 outside the intercalated disc, as an indicator of Cx43 lateralization (*Figure 2B*), reveals a significantly higher percentage in DMD^mdx^ hearts compared with WT hearts, with mean percentages of 37.35 ± 1.70% and 19.07 ± 3.56%, respectively. C271S^+/−^ hearts exhibited values similar (22.77 ± 2.00%) to WT, whereas DMD^mdx^:C271S^+/−^ hearts displayed percentages of Cx43 outside the intercalated disc comparable to DMD^mdx^ hearts (38.99 ± 2.94%) (*Figure 2B*). These findings indicate that lack of S-nitrosylation at residue C271 does not prevent Cx43 lateralization in DMD^mdx^ hearts and suggest that this is not the mechanism by which arrhythmias are ameliorated in DMD^mdx^:C271S^+/−^ mice.

**Figure 2:**
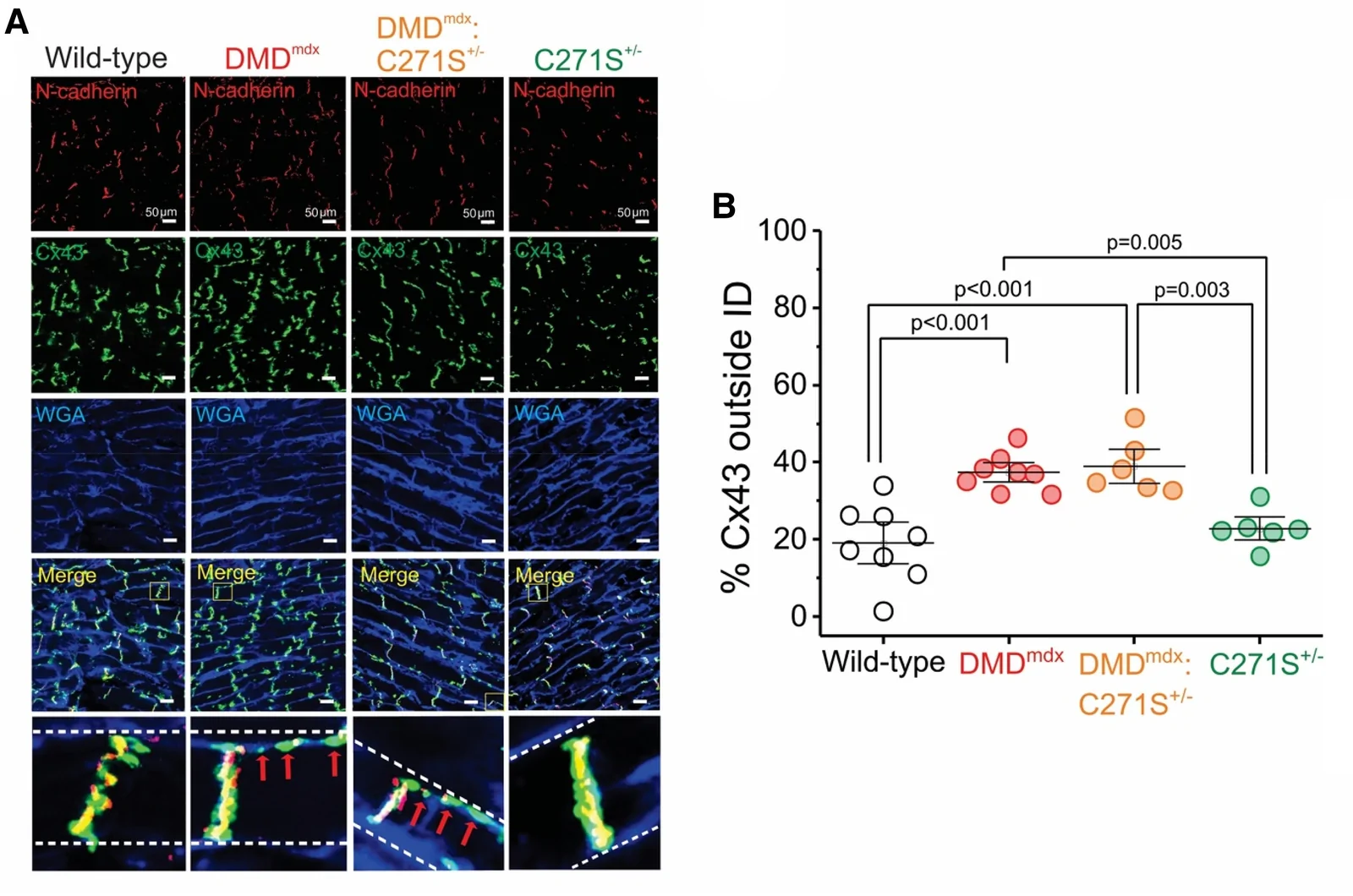
Remodelling of cardiac Cx43 hemichannels is not prevented by decreased levels of S-nitrosylated Cx43 in DMD^mdx^ hearts (DMD^mdx^:C271S^+/−^). (*A*) Representative confocal images of immunofluorescence in the left ventricle against N-cadherin (red) and Cx43 proteins (green) in WT, DMD^mdx^, DMD^mdx^:C271S^+/−^, and C271S^+/−^ mouse hearts. Plasma membranes were stained with WGA (blue). Scale bar = 50 μm. The bottom row shows inset areas (yellow squares) from merged photos of Cx43, N-cadherin (a marker of the intercalated disc), and WGA. Red arrows indicate positive signals for Cx43 at the lateral side of cardiomyocytes (DMD^mdx^ and DMD^mdx^:C271S^+/−^ hearts). (*B*) Percentage of total Cx43 mislocalized outside the intercalated disc, quantified as the fraction of Cx43 signal that does not overlap with N-cadherin relative to the total Cx43 in WT, DMD^mdx^, DMD^mdx^:C271S^+/−^, and C271S^+/−^ mouse heart sections. *n* = 6–8 mice per group. Each dot represents an independent animal. Group comparisons were made using a one-way ANOVA followed by Tukey’s *post-hoc* test. Data are presented as means ± SEM.

### 3.4 Prolonged AP and enhanced Ca^2+^ signalling in DMD^mdx^ hearts are prevented in DMD^mdx^:C271S^+/−^ mice

We previously demonstrated that β-adrenergic stimulation triggers opening of lateralized Cx43 hemichannels, thereby altering cardiac excitability in DMD^mdx^.^19^ Nevertheless, the specific role of Cx43 S-nitrosylation in this process, particularly within the context of the intact heart, remains unexplored. To investigate this, we performed optical mapping to assess membrane potential changes using RH237 in Langendorff-perfused hearts under baseline conditions and during 10 Hz stimulation in the presence of 100 nM isoproterenol at the left ventricles in WT, DMD^mdx^, and DMD^mdx^:C271S^+/−^ mice. *Figure 3A* and *B* show representative heat maps and traces of APDs at 90% repolarization (APD_90_) for WT, DMD^mdx^, and DMD^mdx^:C271S^+/−^ hearts before and during isoproterenol stimulation. APDs were calculated as the average of six independent ROI in the left ventricular area. DMD^mdx^ mice exhibited prolonged APD_90_ at baseline conditions (97.97 ± 9.87 ms) and in the presence of isoproterenol (82.09 ± 4.12 ms) compared with WT mice (baseline_APD90:_ 58.10 ± 3.30 ms; isoproterenol_APD90:_ 53.95 ± 3.34 ms) (*Figure 3C*). Notably, DMD^mdx^:C271S^+/−^ mice demonstrated significantly shorter APDs, resembling those of WT hearts under both baseline conditions and isoproterenol stimulation (baseline_APD90:_ 61.72 ± 4.29 ms, isoproterenol_APD90_: 55.47 ± 1.48 ms) (*Figure 3C*). We further explored how sustained β-receptor overstimulation affects APD by causing prolonged opening of Cx43 hemichannels. Our findings showed that, under these conditions, DMD^mdx^ hearts exhibited prolonged APD_90_, APD_50_, and APD_30_ in the presence of isoproterenol when compared with WT hearts. Importantly, DMD^mdx^:C271S^+/−^ hearts still displayed significantly shorter APDs, similar to those observed in WT hearts (see Supplementary material online, *Figure S5*). These results suggest that S-nitrosylation of Cx43 hemichannels at cysteine 271 is critical for inducing prolonged APs in DMD^mdx^ hearts under cardiac stress.

**Figure 3:**
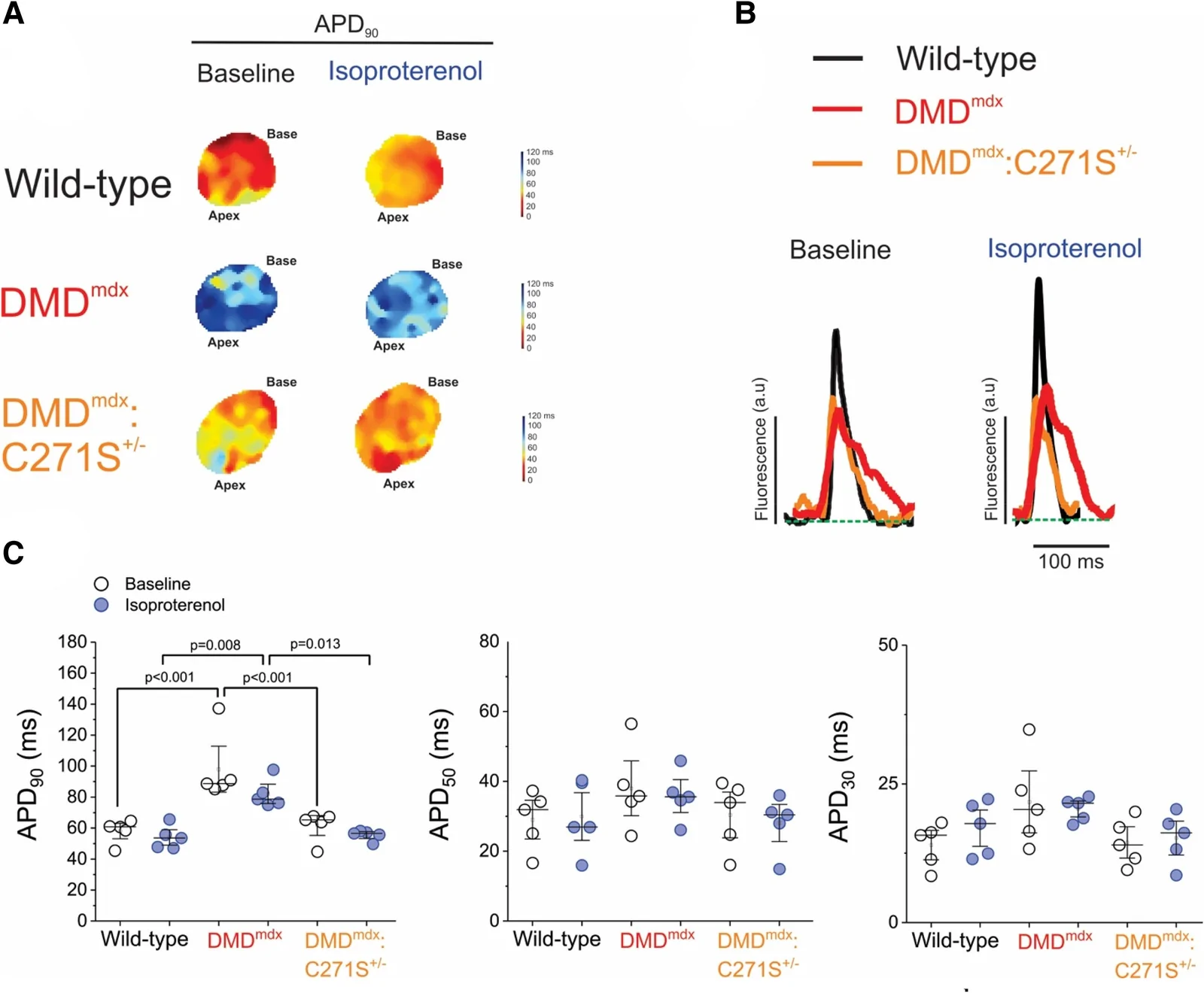
Decreased levels of S-nitrosylated Cx43 hemichannels in DMD^mdx^ mice prevent the prolongation of APD observed in DMD^mdx^ mice. (*A*) Representative heat maps of APD at 90% repolarization (APD_90_) of WT, DMD^mdx^, and DMD^mdx^:C271S^+/−^ hearts before (baseline) and during isoproterenol treatment (100 nM and pacing at 10 Hz). (*B*) Representative optical mapping traces of APDs at baseline conditions and during β-adrenergic stimulation (isoproterenol) in WT (black), DMD^mdx^ (red), and DMD^mdx^:C271S^+/−^ (orange) hearts previously loaded with the voltage-sensitive probe RH237 via the Langendorff perfusion system. (*C*) APDs: APD_90_, APD_50_, APD_30_ before (white circles) and during isoproterenol stimulation (blue circles). APDs were calculated by the average of six independent ROI in the ventricular area of the mouse heart. *n* = 5 mice per group. Each dot represents an independent animal. Group comparisons were made using a two-way ANOVA followed by Tukey’s *post-hoc* test. Data are presented as means ± SEM.

Pathological opening of Cx43 hemichannels has recently been shown to disrupt intracellular Ca^2+^ handling, promoting Ca^2+^ overload in ventricular cardiomyocytes.^22,45,46^ To explore whether cardiac Ca^2+^ signalling is disrupted in DMD and linked to S-nitrosylation of Cx43, we performed optical mapping recordings of Ca^2+^ transients using Rhod-2AM. We evaluated left ventricular Ca^2+^ transients using optical mapping in Langendorff-perfused hearts from WT, DMD^mdx^, and DMD^mdx^:C271S^+/−^ mice under baseline conditions and during 10 Hz stimulation in the presence of 100 nM isoproterenol. *Figure 4A* and *B* show representative transient traces and heat maps of Ca^2+^ amplitudes for WT, DMD^mdx^, and DMD^mdx^:C271S^+/−^ mice before and during isoproterenol stimulation. DMD^mdx^ mice exhibited significantly larger post-isoproterenol/baseline ratios for Ca^2+^ transient amplitude and Ca^2+^ decay constant (1.96 ± 0.11 and 1.56 ± 0.25, respectively) than WT hearts (1.32 ± 0.01 and 0.61 ± 0.09, respectively) and DMD^mdx^:C271S^+/−^ hearts (1.34 ± 0.04 and 0.66 ± 0.07, respectively) (*Figure 4C*). The individual Ca^2+^ decay constant values before isoproterenol stimulation were 31.02 ± 2.13 ms in WT hearts, 44.40 ± 4.29 ms in DMD^mdx^ hearts, and 31.46 ± 2.78 ms in DMD^mdx^:C271S^+/−^ hearts. Following isoproterenol stimulation, the individual Ca^2+^ decay constant values were 18.47 ± 1.94 ms in WT hearts, 67.26 ± 9.06 ms in DMD^mdx^ hearts, and 20.49 ± 1.75 ms in DMD^mdx^:C271S^+/−^ hearts (*Figure 4C*). We also found that sustained β-receptor overstimulation resulted in similar Ca^2+^ transient amplitudes and decay constants, but with a substantial lack of chronotropic response (see Supplementary material online, *Figure S6*). Overall, these findings suggest that S-nitrosylation of Cx43 hemichannels disrupts intracellular cardiac Ca^2+^ signalling following β-adrenergic stimulation in DMD^mdx^ mice, potentially contributing to the development of cardiomyopathies.

**Figure 4:**
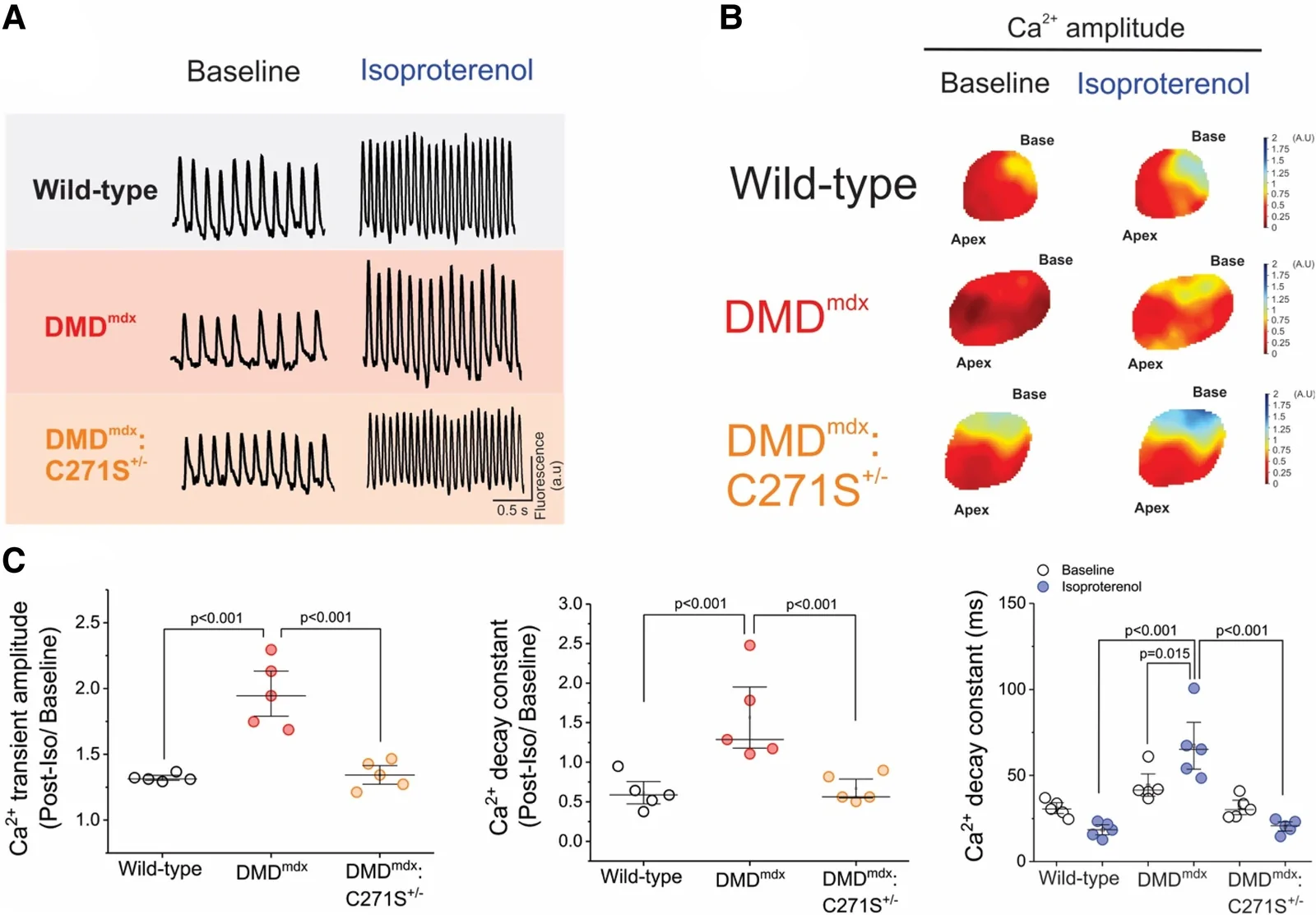
S-nitrosylation of cardiac Cx43 hemichannels in DMD^mdx^ mice mediates increased cardiac Ca^2+^ signalling. (*A*) Representative Ca^2+^ traces under baseline conditions and during β-adrenergic stimulation with 100 nM isoproterenol and pacing at 10 Hz in WT (black), DMD^mdx^ (red), and DMD^mdx^:C271S^+/−^ (orange) in hearts previously loaded with the Ca^2+^ indicator Rhod-2 via Langendorff perfusion system. (*B*) Representative heat maps of Ca^2+^ amplitudes of WT, DMD^mdx^, and DMD^mdx^:C271S^+/−^ hearts before (baseline) and during isoproterenol treatment. (*C*) Quantification of Ca^2+^ transient amplitudes ratios (left panel), decay constant ratios (middle panel), and individual values (right panel) before and post-β-adrenergic stimulation with isoproterenol. Ca^2+^ amplitudes were calculated as the average of six independent ROI in the ventricular area of the mouse heart. *n* = 5 mice per group. Each dot represents an independent animal. Group comparisons were made using a one-way ANOVA followed by Tukey’s *post-hoc* test. Data are presented as means ± SEM.

### 3.5 S-nitrosylation of cardiac Cx43 hemichannels in DMD^mdx^ induces diastolic interval heterogeneity in response to frequency and β-adrenergic stimulation

Under pathological conditions, repeated electrical stimulation may disrupt cardiac excitability and Ca^2+^ handling. To examine this effect and the role of S-nitrosylation of Cx43 hemichannels, we performed dual optical mapping of membrane voltage and intracellular Ca^2+^ in Langendorff-perfused hearts from WT, DMD^mdx^, and DMD^mdx^:C271S^+^/^−^ mice. We quantified ventricular AP properties and Ca^2+^ transient parameters at baseline, during 10 Hz electrical stimulation delivered at the ventricular apex in the presence of 100 nM isoproterenol, and after a 2-min recovery period during spontaneous sinus rhythm. *Figure 5A* shows representative images of APDs for WT, DMD^mdx^, and DMD^mdx^:C271S^+/−^ hearts during baseline, 10 Hz stimulation with 100 nM isoproterenol, and 2 min post-stimulation. WT and DMD^mdx^:C271S^+/−^ hearts exhibited consistent rhythmicity in APDs during baseline, electrical stimulation with β-adrenergic activation, and the 2-min recovery phase. In contrast, DMD^mdx^ hearts displayed reduced signal amplitudes in APs at baseline, as well as altered responsiveness to electrical and isoproterenol stimulation, and 2 min after stimulation. WT, DMD^mdx^, and DMD^mdx^:C271S^+/−^ hearts exhibited an increase in heart rate following isoproterenol stimulation, with a significant elevation from baseline values (WT, DMD^mdx^, and DMD^mdx^:C271S^+/−^; 295.52 ± 10.34, 277.37 ± 30.97, and 332.04 ± 38.40 beats per minute, respectively) to isoproterenol-treated conditions (WT, DMD^mdx^, and DMD^mdx^:C271S^+/−^: 518.85 ± 32.32, 545.05 ± 39.09, and 525.85 ± 20.88 beats per minute, respectively) (*Figure 5B*). Two minutes post-isoproterenol stimulation, heart rates remained elevated in all groups, with values of 436.55 ± 26.57, 415.80 ± 27.65, and 534.72 ± 28.57 beats per minute for WT, DMD^mdx^, and DMD^mdx^:C271S^+/−^ hearts, respectively. Two minutes post-stimulation, DMD^mdx^ hearts exhibited episodes of conduction block (*Figure 5A*). To quantify these observations, we calculated the COV for diastolic intervals across beats. Diastolic intervals were determined using MappingLab software, which measures the time from APD_90_ to the activation time of the subsequent AP^36^ (*Figure 5C*). In DMD^mdx^ hearts, the percentage of COV after 2 min of repeated β-adrenergic stimulation (19.94 ± 3.66%) was significantly higher compared with baseline conditions (13.92 ± 3.10%) and during stimulation (5.66 ± 1.93%). In contrast, WT and DMD^mdx^:C271S^+/−^ hearts exhibited similar percentages of COV post-stimulation (0.79 ± 0.21% and 2.03 ± 0.53%, respectively) (*Figure 5D*), suggesting a comparable level of heterogeneity in diastolic intervals across both conditions. Under conditions of sustained β-adrenergic stimulation, similar effects were observed (see Supplementary material online, *Figure S7*). Consistent with the conduction block observed in intact DMD^mdx^ hearts, isoproterenol markedly increased TA in isolated DMD^mdx^ cardiomyocytes (from 3.19 ± 1.05 to 75.94 ± 14.57 events/min), whereas DMD^mdx^:C271S^+/−^ cells remained largely unaffected (1.11 ± 0.54 to 3.63 ± 2.4 events/min; Supplementary material online, *Figure S8*). Together, these findings indicate that S-nitrosylation-dependent activation of Cx43 hemichannels promotes conduction block, TA, and Ca^2+^ dysregulation in DMD^mdx^ hearts, consistent with the arrhythmogenic phenotype observed *in vivo*.

**Figure 5:**
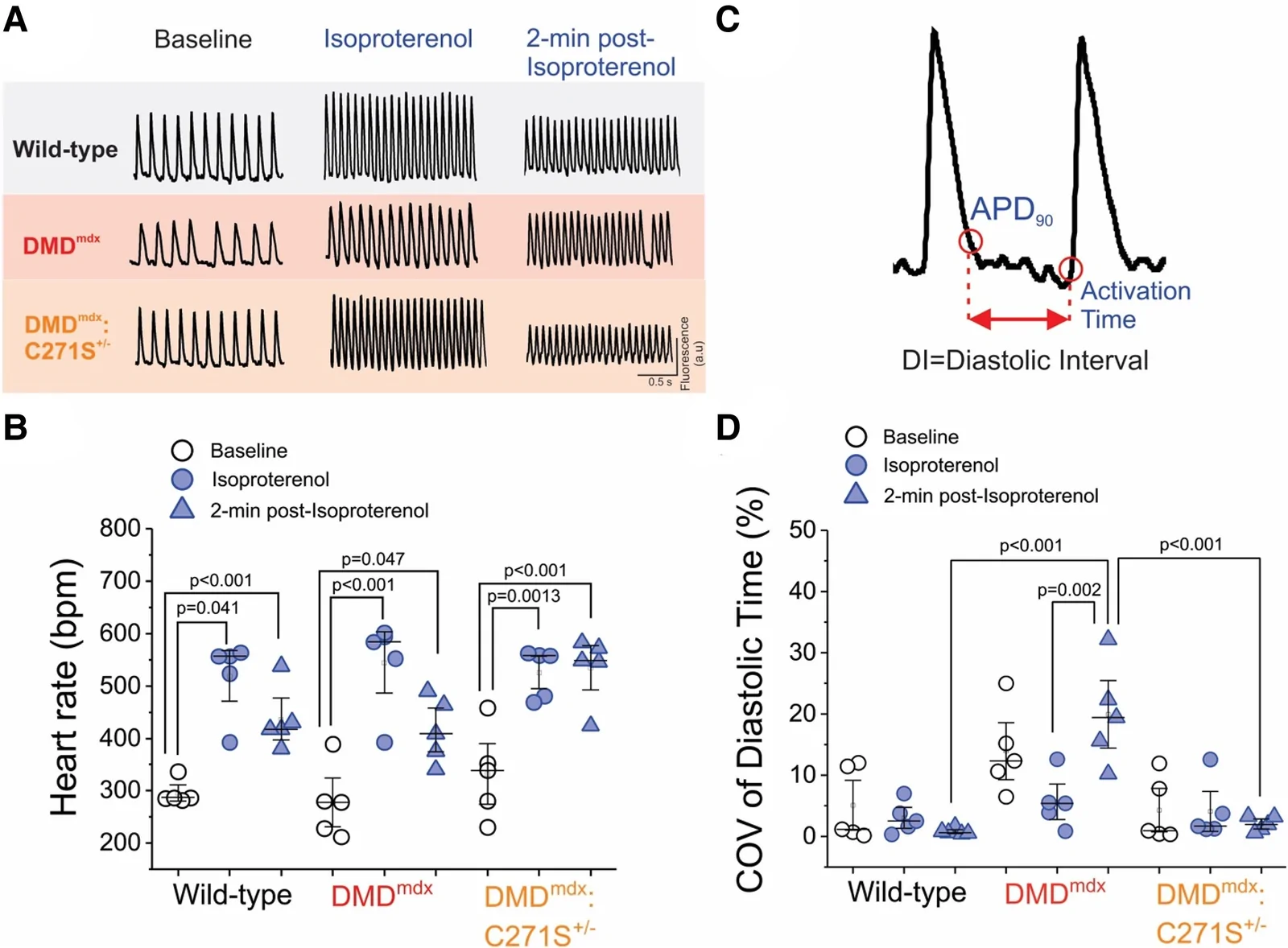
S-nitrosylation of cardiac Cx43 hemichannels in DMD^mdx^ mice promotes diastolic interval heterogeneity via frequency stimulation and β-adrenergic-mediated cardiac stress. (*A*) Representative optical mapping traces of AP recorded from WT (black), DMD^mdx^ (red), and DMD^mdx^:C271S^+/−^ (orange) hearts at baseline conditions (left), at 10 Hz burst pacing in the presence of 100 nM isoproterenol (middle), or after recovery (2-minutes post-isoproterenol). Scale bar: 0.5 s. (*B*) Quantification of heart rate at baseline (left), during (middle), and 2 min (right) after 10 Hz burst pacing + 100 nM of Iso stimulation. (*C*) Schematic representation of the diastolic interval, defined as the time from APD_90_ of an AP to the activation of the subsequent AP, as previously described.^36,37^ (*D*) Average COV (as a percentage) of diastolic intervals between cardiac APs at baseline (white circles), 10 Hz + Iso (blue circles), and 2 min after electrical stimulation (blue triangles) in WT, DMD^mdx^, and DMD^mdx^:C271S^+/−^ hearts. The COV of diastolic intervals was calculated as the standard deviation divided by the mean diastolic interval. Diastolic intervals were measured from APD_90_ to the activation time of the subsequent AP, averaged over six consecutive beats from six independent ROI within the ventricular area of the heart. *n* = 5 mice per group. Each data point represents an individual animal. Statistical comparisons were performed using a two-way ANOVA followed by Tukey’s *post-hoc* test. Data are presented as mean ± SEM.

### 3.6 Gap19-mediated inhibition of Cx43 hemichannels prevents β-adrenergic–induced diastolic interval instability, APD_90_ prolongation, and abnormal Ca^2+^ handling in DMD^mdx^ hearts

To further confirm whether Cx43 hemichannel activity contributes to β-adrenergic–induced electrical instability in DMD^mdx^ hearts, we assessed the effects of acute blockade with the selective inhibitor Gap19. DMD^mdx^ mice received vehicle or Gap19 (10 mg/kg) via retro-orbital injection 1–2 h before heart isolation and hearts were subsequently studied *ex vivo* by optical mapping. Isoproterenol increased heart rate similarly in vehicle and Gap19-treated DMD^mdx^ hearts (245.6 ± 18.4 to 548.8 ± 38.5 bpm vs. 259.3 ± 28.3 to 544.7 ± 17.8 bpm; *Figure 6B*), indicating preserved chronotropic responsiveness. However, vehicle-treated DMD^mdx^ hearts developed marked post-stimulation electrical instability, with diastolic interval variability increasing from 7.72 ± 3.70% to 15.54 ± 3.69%, whereas Gap19-treated hearts remained stable (0.98 ± 0.53% to 2.49 ± 1.32%; *Figure 6C*). As expected, APD_90_ remained prolonged in DMD^mdx^ hearts (82.18 ± 3.83, 66.53 ± 2.32, 52.90 ± 6.46 ms; baseline, Iso, recovery) compared with Gap19-treated hearts (56.95 ± 2.59, 43.47 ± 2.49, 46.30 ± 4.67 ms; *Figure 6D*). Gap19 also attenuated Ca^2+^ dysregulation, reducing the increase in Ca^2+^ transient amplitude (1.69 ± 0.11 vs. 1.31 ± 0.05, post-Iso/baseline ratio; *Figure 6E*) and normalizing decay kinetics (decay constant ratio: 1.06 ± 0.09 vs. 0.73 ± 0.05; *Figure 6F*). Baseline and post-stress decay constants were also improved (33.19 ± 5.14 to 33.68 ± 4.04 ms vs. 39.12 ± 3.86 to 28.65 ± 3.87 ms, *Figure 6G*). Collectively, these data indicate that Cx43 hemichannels drive β-adrenergic–induced diastolic instability, APD prolongation, TA, and Ca^2+^ dysregulation in DMD^mdx^ myocardium. Gap19 preserves chronotropic responses while preventing these abnormalities, supporting Cx43 hemichannels as a putative therapeutic target.

**Figure 6:**
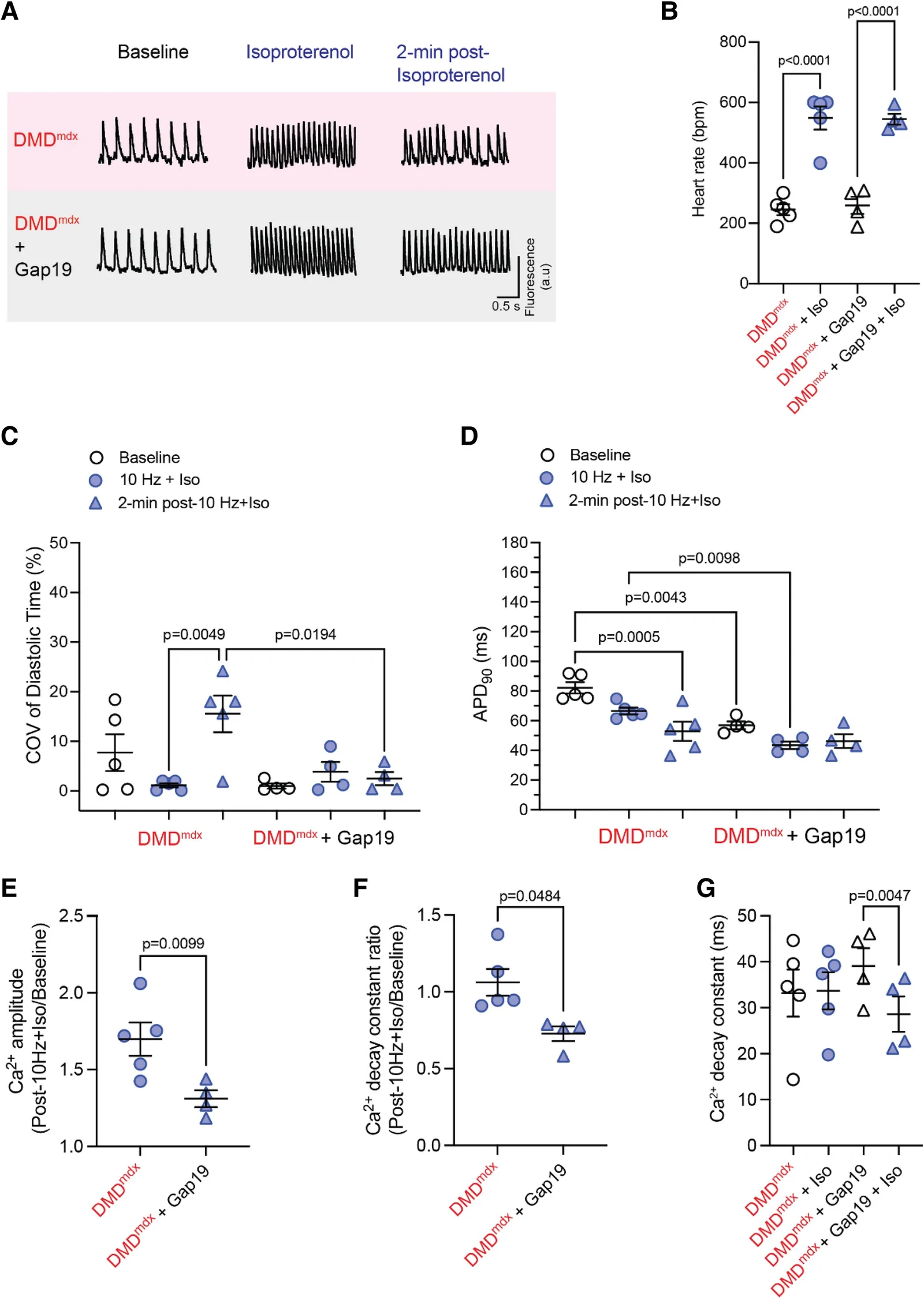
Gap19-mediated inhibition of cardiac Cx43 hemichannels suppresses β-adrenergic stress–induced diastolic interval heterogeneity and increased Ca^2+^ signalling in DMD^mdx^ hearts. (*A*) Representative optical mapping recordings of APs from DMD^mdx^ hearts at baseline (left), during isoproterenol stimulation (middle), and after recovery (2 min post-isoproterenol; right), in the absence or presence of the Cx43 hemichannel blocker Gap19. Gap19 was delivered by retro-orbital injection 1 h before optical mapping recordings, ensuring cardiac exposure before functional measurements. Scale bar: 0.5 s. (*B*) Quantification of heart rate at baseline and during isoproterenol stimulation in DMD^mdx^ hearts in the absence or presence of Gap19. (*C*) Average COV (as a percentage) of diastolic intervals between cardiac APs at baseline (white circles), during 10 Hz stimulation in the presence of isoproterenol (blue circles), and 2 min after stimulation (blue triangles) in DMD^mdx^ hearts in the absence or presence of Gap19. The COV of diastolic intervals was calculated as the standard deviation divided by the mean diastolic interval. (*D*) Quantification of APD_90_ at baseline (white circles), during 10 Hz+ isoproterenol stimulation (blue circles), and 2 min after stimulation (blue triangles) in DMD^mdx^ hearts in the absence or presence of Gap19. (*E*) Quantification of Ca^2+^ transient amplitude, expressed as post-10 Hz+ isoproterenol/baseline ratio, in optical mapping recordings from DMD^mdx^ and DMD^mdx^ + Gap19 hearts. Gap19 was administered by retro-orbital injection 1 h before heart isolation and optical mapping recordings. (*F*) Quantification of Ca^2+^ decay constant, expressed as post-10 Hz+ isoproterenol/baseline ratio, in optical mapping recordings from DMD^mdx^ and DMD^mdx^ + Gap19 hearts. (*G*) Quantification of decay tau under baseline conditions and after 10 Hz pacing plus isoproterenol in DMD^mdx^ and DMD^mdx^ + Gap19 hearts. *n* = 4–5 mice per group. Group comparisons were made using an unpaired *t*-test in (*E*) and (*F*), and a two-way ANOVA followed by Tukey’s *post-hoc* test in G. Each data point represents an individual heart (*n* = 4–5). Data are presented as mean ± SEM.

### 3.7 DMD^mdx^:C271S^+/−^ mice are protected against bradycardia and myocardial injury induced by isoproterenol stimulation

Multiple reports describe myocardial injury in DMD patients without clear aetiological mechanisms.^4,5,47^ Given our previous findings that blocking Cx43 hemichannels reduces lethality in DMD^mdx^ mice following cardiac stress,^19^ we investigated whether S-nitrosylation-dependent Cx43 hemichannel opening serves as a critical link between myocardial injury and lethal arrhythmias. Telemetric ECG measurements revealed that DMD^mdx^ mice developed abnormal bradycardia after 4 h post-isoproterenol treatment. Given that bradycardia contributes to the onset of life-threatening ventricular tachycardia in animal models,^48^ we investigated whether this effect could be prevented by blocking Cx43 hemichannels or disrupting Cx43 S-nitrosylation. *Figure 7A* shows that DMD^mdx^ mice exhibited significantly lower heart rates after isoproterenol injection (Heart rate 6h−, 9h-, and 14h-post-Iso: 309.59 ± 28.14 beats/min, 351.28 ± 45.88 beats/min, 345.47 ± 60.55 beats/min) when compared with WT mice (Heart rate 6h-, 9h-, and 14h-post-Iso: 523.12 ± 26.94 beats/min, 539.93 ± 23.66 beats/min, and 537.14 ± 18.19 beats/min, respectively). Notably, DMD^mdx^:C271S^+/−^ (Heart rate 6h-, 9h-, and 14h-post-Iso: 517.90 ± 65.87, 571.48 ± 35.90, and 543.82 ± 36.46 beats/min, respectively) or DMD^mdx^ mice treated with Gap19 (14 h post-Iso: 595.36 ± 14.89) displayed similar heart rates to those found in WT mice several hours after isoproterenol (*Figure 7B*). These findings suggest that the opening of S-nitrosylated Cx43 hemichannels plays a critical role in the delayed onset of bradycardia observed hours after cardiac stress in DMD^mdx^ mice.

**Figure 7:**
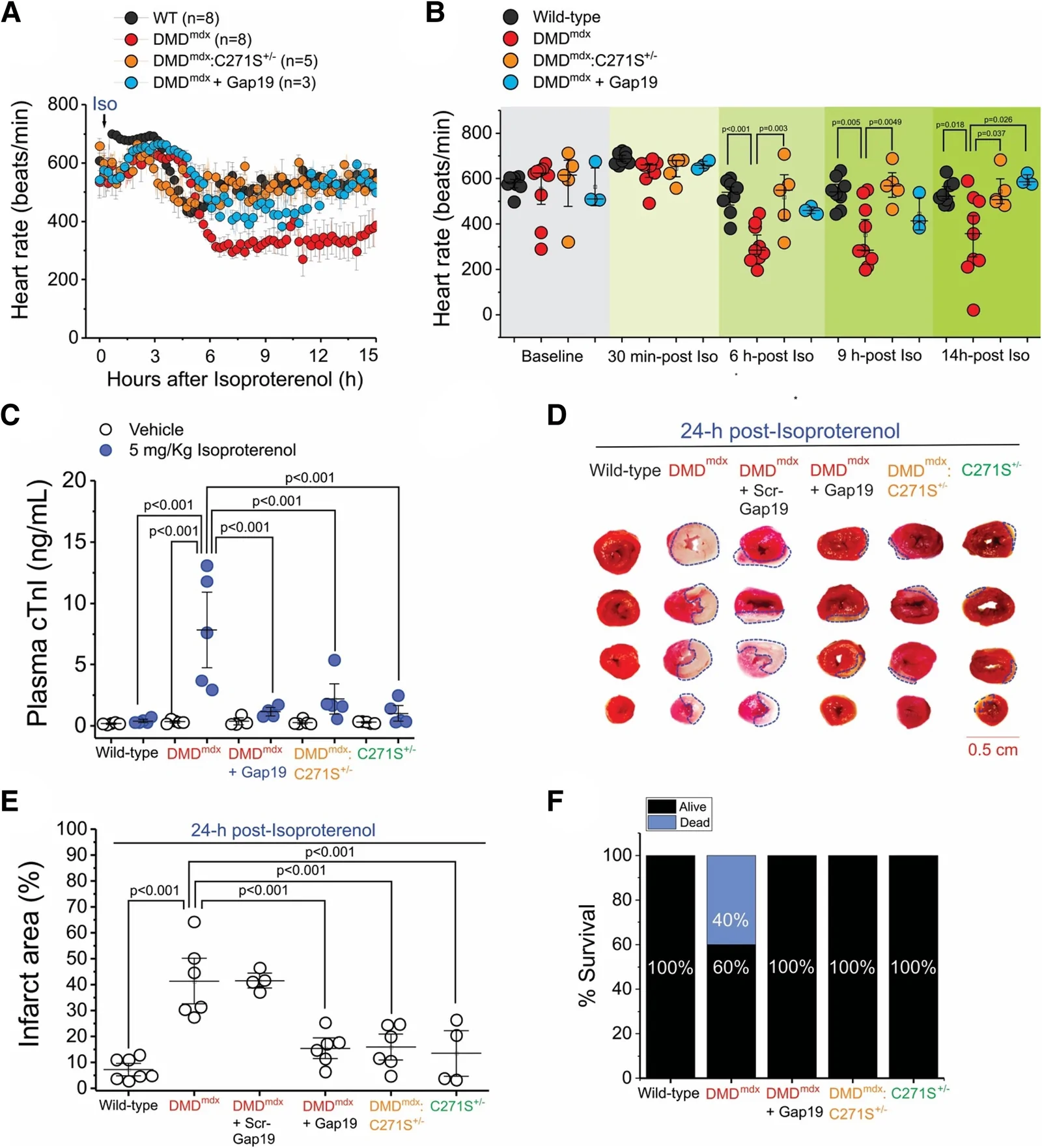
Reduced S-nitrosylated cardiac Cx43 hemichannels attenuate bradycardia and myocardial injury after β-adrenergic stress. (*A*) Representative heart rate traces from freely moving WT, DMD^mdx^, DMD^mdx^ mice treated with Gap19 (10 mg/kg, retro-orbital injection), and DMD^mdx^: C271S^+/−^ mice, recorded via ECG telemetry before and after IP isoproterenol administration (5 mg/kg). Heart rate analysis was performed considering the surviving mice after isoproterenol stimulation. (*B*) Average heart rate values were measured at baseline and 30 min, 6 h, 9 h, and 14 h post-isoproterenol administration using ECG telemetry implants. (*C*) Plasma cTnI levels 4 h after isoproterenol-mediated cardiac stress in WT, DMD^mdx^, DMD^mdx^ treated with Gap19 via retro-orbital injection (10 mg/kg), DMD^mdx^:C271S^+/−^, and C271S^+/−^ mice. Group comparisons were made using a two-way ANOVA followed by Tukey’s *post-hoc* test. (*D*) Representative images of cross-sections of mouse ventricles stained with TTC 24 h post-isoproterenol stimulation. Myocardial injury was outlined by a blue line. Scale bar: 0.5 cm. (*E*) Quantification of % + TTC area of heart ventricles after β-adrenergic stimulation with respect to the total slice areas in WT, DMD^mdx^, DMD^mdx^ previously treated with Gap19 via retro-orbital injection (10 mg/kg), DMD^mdx^ treated with the scrambled Gap19 peptide (Scr-Gap19, sequence: IKFKKEIKQ, 10 mg/kg) via retro-orbital injection, DMD^mdx^:C271S^+/−^, and C271S^+/−^ mice. Group comparisons were made using a one-way ANOVA followed by Tukey’s *post-hoc* test. (*F*) Percentage of survival of WT, DMD^mdx^, DMD^mdx^ treated with Gap19 via retro-orbital injection (10 mg/kg), DMD^mdx^:C271S^+/−^, and C271S^+/−^ mice 24 h post-isoproterenol stimulation. Each dot represents an independent mouse. *n* = 3–8 mice per group. Data are presented as means ± SEM.

Both DMD patients and DMD mouse models also exhibit elevated levels of cTnI, which is associated with myocardial injury and the development of cardiomyopathies throughout life.^6,26–29^ Thus, we investigated whether decreased levels of S-nitrosylated Cx43 prevent elevated cTnI levels in DMD^mdx^ mice under cardiac stress, using a high-sensitivity cTnI ELISA Kit (*Figure 7C*). We observed that plasma levels of cTnI significantly increased 4 h post-isoproterenol treatment in DMD^mdx^ mice (7.83 ± 2.05 ng/mL) compared with baseline conditions (0.34 ± 0.06 ng/mL). Upon β-adrenergic stimulation, DMD^mdx^ mice treated with Gap19 (1.15 ± 0.22 ng/mL), DMD^mdx^:C271S^+/−^ (2.20 ± 0.82 ng/mL), C271S^+/−^ (0.79 ± 0.42 ng/mL), and WT mice (0.40 ± 0.08 ng/mL) showed considerably lower levels of plasma cTnI, similar to the baseline levels of each experimental condition (*Figure 7C*). Following 24 h of isoproterenol stimulation, myocardial injury was assessed using the TTC staining in 2-mm ventricular heart sections to evaluate whether S-nitrosylated Cx43 hemichannels are involved in ventricular myocardial injury in DMD^mdx^ mice under β-adrenergic stimulation. Isoproterenol-treated hearts from DMD^mdx^ (41.33 ± 5.85%) and the scrambled Gap19 peptide (41.54 ± 1.91%) mice exhibited larger areas of myocardial injury compared with WT conditions (7.17 ± 1.58%) after 24 h of cardiac stress. Strikingly, there were no significant differences in infarct areas between DMD^mdx^ mice previously treated retro-orbitally with the Cx43 hemichannel blocker, Gap19 (10 mg/kg) (15.38 ± 2.62%), DMD^mdx^:C271S^+/−^ (15.90 ± 3.36%), C271S^+/−^ (13.40 ± 5.87%), and WT mice (7.17 ± 1.58%) (*Figure 7D* and *E*). Consistent with these results, DMD^mdx^ mice exhibited ∼40% mortality after 24 h post-isoproterenol stimulation, which was prevented in DMD^mdx^:C271S^+/−^ mice, indicating that reduced levels of S-nitrosylation of Cx43 are sufficient to rescue the survival percentage observed in WT conditions (100% survival in mouse models of DMD^mdx^:C271S^+/−^ knock-in colonies) (*Figure 7F*). These findings indicate that lateralized S-nitrosylation of Cx43 hemichannels critically contributes to bradycardia and myocardial injury following β-adrenergic receptor activation in DMD^mdx^ mice.

## 4. Discussion

Despite growing evidence implicating lateralized Cx43 hemichannels as key pathological drivers in DMD cardiomyopathies,^18–20^ the fundamental mechanisms underlying their hyperactivity remain elusive.^19^ In this study, we evaluated the pathological role of Cx43 S-nitrosylation at cysteine 271, evoking deadly arrhythmias and myocardial injury after β-adrenergic-evoked cardiac stress. We found that β-adrenergic stimulation in DMD^mdx^ provokes severe arrhythmias, aberrant Ca^2+^ signalling, prolonged APD, late-onset bradycardia, and myocardial injury, but these pathological features are prevented in DMD^mdx^:C271S^+/−^ mice with impaired Cx43 S-nitrosylation. These findings establish Cx43 S-nitrosylation at cysteine 271 as a crucial molecular mechanism underlying β-adrenergic-driven cardiac dysfunction in DMD cardiomyopathy.

DMD is associated with an autonomic nervous system imbalance, characterized by reduced parasympathetic activity and heightened sympathetic drive.^49,50^ The pathological cardiac features associated with DMD include elevated heart rates, tachycardia, and an increased risk of sudden cardiac death.^7,49,51^ Similarly, the DMD^mdx^ mouse model exhibits tachycardia, reduced heart rate variability, and autonomic dysfunction, mirroring the parasympathetic suppression and sympathetic overactivation observed in patients. These features make DMD^mdx^ a valuable mouse model for investigating the mechanisms underlying DMD cardiomyopathy.^51^ Since cardiac pathology in DMD^mdx^ mice generally develops after 8 months of age, 4- to 6-month-old mice reflect an early disease stage in which β-adrenergic challenge can reveal underlying cardiac vulnerability before progression to advanced DMD cardiomyopathy.^19,52^ In DMD^mdx^ mice, we have previously shown that β-adrenergic–induced arrhythmias and sudden death are primarily mediated by the hyperactivity of remodelled (lateralized) Cx43 hemichannels.^18,19^ While the mechanisms underlying lateralization have been partially unveiled, the opening of these hyperactive hemichannels remains less understood.

Cx43 remodelling has been long associated with cardiac dysfunction in multiple models of heart failure,^53–55^ myocarditis,^56^ ischaemia,^17,57^ arrhythmogenic cardiomyopathy,^58^ and DMD^mdx^ cardiomyopathy.^19,20^ This lateralization results from reduced post-translational phosphorylation at C-terminal serine S325/S328/S330 in dystrophic human samples and mouse DMD^mdx^ hearts via a microtubule-dependent reorganization mechanism.^18,59^ Importantly, DMD^mdx^:C271S^+/−^ mice, which have impaired Cx43 S-nitrosylation, displayed similar Cx43 protein lateralization as observed in DMD^mdx^ heart sections. This result indicates that S-nitrosylation is not a post-translational modification involved in Cx43 cardiac lateralization. Therefore, the cardiac protection observed in DMD^mdx^:C271S^+/−^ mice is linked to reduced or blunted opening of Cx43 hemichannels rather than the prevention of remodelling. This aligns with the heterologous characterization of the Cx43 C271S mutation, which exerts a dominant-negative effect on WT Cx43 hemichannel activity *in vitro*.

β-adrenergic signalling promotes NO production in cardiomyocytes, leading to S-nitrosylation of key Ca^2+^-handling proteins, including RyRs, SERCA2-associated phospholamban, CaMKII, sodium–calcium exchanger (NCX), and troponin C.^34,60–62^ In DMD hearts, nNOS and eNOS are down-regulated, while iNOS is up-regulated, producing excessive NO that disrupts redox balance and may contribute to arrhythmias,^31,63–67^ suggesting that iNOS, along with dysregulated NOS isoforms, constitutes a putative source of aberrant NO signalling in DMD pathology. We previously showed that L-NAME treatment reduces Cx43 S-nitrosylation in DMD^mdx^ mice, mitigating Cx43-mediated excitability dysfunction and arrhythmogenicity.^19^ However, because L-NAME affects multiple cardiac proteins, its direct impact on the S-nitrosylation and activity of Cx43 hemichannels remained unclear. Our current work demonstrates that isoproterenol-induced arrhythmias and TA are significantly reduced in DMD^mdx^:C271S^+/−^ mice, confirming that S-nitrosylation of Cx43 hemichannels plays a direct role in life-threatening arrhythmias. This is the first study to identify cysteine 271 in Cx43 as a critical regulatory site for hemichannel activity *in vivo*, highlighting its potential as a cardiac therapeutic target.

Our published data demonstrated that isoproterenol induces membrane depolarization and TA in isolated DMD^mdx^ cardiomyocytes.^19^ We also found that DMD^mdx^ cardiomyocytes displayed longer APDs with respect to WT cardiomyocytes. These changes in membrane excitability were prevented in the presence of Cx43 hemichannel blockers (Gap19 or the antibody against Cx43) or the generic inhibitor of nitric oxide synthase, L-NAME.^19^ Similar results were found in isolated cardiomyocytes from a genetically modified Cx43 knock-in mouse (S3A) that promotes cardiac remodelling of Cx43 protein without apparent cardiac dysfunction.^22^ While electrophysiology in isolated cardiomyocytes is informative regarding Cx43 hemichannel activity,^22^ there is always the concern that cell isolation *per se* might increase remodelling of Cx43 hemichannels. For this reason, we used optical mapping in isolated mouse hearts, which allowed us to examine the role of Cx43 proteins in a preparation where the functional and structural integrity of the myocardium is preserved. We consistently found that DMD^mdx^ mice exhibited prolonged APD at baseline, which remained extended in the presence of isoproterenol, whereas no such prolongation was observed in DMD^mdx^:C271S^+/−^ hearts. As expected, DMD^mdx^:C271S^+/−^ hearts displayed shorter APDs under baseline conditions and in response to isoproterenol, similar to WT conditions. This suggests that S-nitrosylation of Cx43 plays a critical role in modulating cardiac excitability during the late phases of repolarization, as reflected by APD_90_. Under conditions of exacerbated β-adrenergic stimulation, our data suggest that S-nitrosylation of Cx43 is also important in both the early (APD_30_ and APD_50_) and late phases of repolarization (APD_90_) (see Supplementary material online, *Figure S5*); likely due to the opening of Cx43 hemichannels. Excessive β-adrenergic stimulation has been shown to profoundly disrupt myocardial integrity, exacerbating oxidative stress, inducing hypertrophy, and altering intracellular signalling pathways mediated by G protein-coupled receptor activation.^68^ Notably, chronic isoproterenol treatment has been associated with β-receptor desensitization, leading to diminished cAMP formation and a reduced contractile response to catecholamines.^69^ This desensitization may explain the absence of a chronotropic effect observed when hearts were stimulated at µM concentrations of isoproterenol, as well as the lack of chronotropic responsiveness (see Supplementary material online, *Figure S6*). Furthermore, the pronounced β-adrenergic overstimulation appears to influence early phases of repolarization, as reflected by alterations in APD_30_ and APD_50_. These findings suggest that excessive β-receptor activation not only compromises Ca^2+^ handling but also disrupts electrophysiological properties, potentially contributing to arrhythmogenic vulnerability in dystrophic hearts. S-nitrosylation of Cx43 hemichannels in a whole-heart preparation appears to influence local membrane potentials, helping to maintain prolonged APs during β-adrenergic stimulation without extending them beyond baseline levels. These electrophysiological parameters could serve as early markers of cardiac pathology during various stages of the repolarization phase of the cardiac AP.

Isolated dystrophic cardiomyocytes also exhibit abnormal intracellular Ca^2+^ signalling^63^; however, the mechanisms underlying this dysregulation are not yet fully understood.^45^ Consistently, our data showed that DMD^mdx^ hearts show increased Ca^2+^ levels in the left ventricle during optical mapping experiments following β-adrenergic stimulation with isoproterenol. One proposed mechanism for Ca^2+^ mishandling involves structural and functional remodelling of ryanodine receptors in the DMD^mdx^ model. Leaky RyR2 and diastolic sarcoplasmic reticulum Ca^2+^ leak have been shown to mediate aberrant depolarization of isolated cardiomyocytes, leading to arrhythmias *in vivo.*^63^ Interestingly, Cx43 colocalizes with large dyadic RyR superclusters, forming microdomains at the perinexus.^45,70^ It has also been reported that Ca^2+^ release from the sarcoplasmic reticulum opens Cx43 hemichannels in isolated mouse and pig left ventricular cardiomyocytes under WT conditions, as well as in human heart failure.^45^ Consistently, DMD^mdx^:C271S^+/−^ hearts show only a slight increase in Ca^2+^ transient amplitude and similar Ca^2+^ decay constants to those found in WT hearts. This supports the idea that S-nitrosylation of Cx43 hemichannels mediates Ca^2+^ dysfunction in dystrophic cardiomyocytes.

Our findings indicate that Cx43 hemichannels act not only as effectors of membrane depolarization but also as important contributors to Ca^2+^ dysregulation in dystrophic myocardium. In DMD^mdx^ hearts, β-adrenergic stress was associated with increased Ca^2+^ transient amplitude, impaired Ca^2+^ decay, diastolic interval instability, APD_90_ prolongation, and marked TA, whereas these abnormalities were attenuated either by Cx43 hemichannel blockade with Gap19 or by reducing Cx43 S-nitrosylation in DMD^mdx^:C271S^+/−^ cardiomyocytes. Importantly, L-type Ca^2+^ current (I_Ca,L_) density was unchanged across WT, DMD^mdx^, and DMD^mdx^:C271S^+/−^ cardiomyocytes (see Supplementary material online, *Figure S9*), indicating that the protective phenotype is not attributable to alterations in L-type Ca^2+^ influx. Instead, these data support pathological Cx43 hemichannel opening as an upstream mechanism linking β-adrenergic stress to abnormal Ca^2+^ cycling and arrhythmogenic remodelling. One plausible mechanism is that aberrant hemichannel activity disrupts ionic homeostasis, secondarily impairing intracellular Ca^2+^ handling and promoting repolarization heterogeneity and TA. Consistent with this, Gap19 preserved chronotropic responsiveness while reducing both electrical and Ca^2+^-handling abnormalities, highlighting Cx43 hemichannels as a therapeutically actionable target in DMD-associated arrhythmogenesis.

Interestingly, the activation of Cx43 hemichannels has been suggested to be modulated by frequency and β-adrenergic stimulation, similar to other channels such as RyRs, which are dependent on Ca^2+^/calmodulin-dependent kinase II (CaMKII) activation during high-frequency stimulation at the dyad in the T-tubules.^45,71,72^ The open probability of Cx43 channels has been shown to increase proportionally at frequencies of 0.5 and 4 Hz in mouse, and 0.5 to 2 Hz in pig ventricular cardiomyocytes.^45^ Correspondingly, our data from mouse hearts indicate that high-frequency stimulation approaching 10 Hz, which mimics cardiac stress under a catecholaminergic challenge,^73^ combined with isoproterenol stimulation, enhances AP heterogeneity and the amplitude of intracellular Ca^2+^ signalling, thus augmenting the heterogeneity of diastolic intervals among APs on a beat-to-beat basis (*Figure 5* and Supplementary material online, *Figure S7*). Our results reveal that the percentage COV in diastolic time does not increase during high-frequency stimulation but does so during the recovery period, 2 min after high-frequency and adrenergic stimulation (*Figure 5* and Supplementary material online, *Figure S7*). This suggests that the recovery phase after tachyarrhythmic events plays a crucial role in mediating the activation of Cx43 proteins, thereby contributing to the disruptions in Ca^2+^ homeostasis and APs, and to the generation of life-threatening arrhythmias.

DMD patients have been reported to exhibit episodes of bradycardia^74–77^; however, until now, this phenomenon has not been described in murine models following β-adrenergic stimulation, largely due to the absence of telemetric studies in these animals. Remarkably, we provide the first evidence that cardiac Cx43 proteins contribute to pathological bradycardia several hours post-isoproterenol administration (*Figure 7*), a finding that represents a paradigm shift in our understanding of Cx43-mediated dysregulation in dystrophic hearts. This delayed bradycardic response may be a consequence of myocardial injury or ischaemic-like damage, ultimately predisposing to lethal ventricular arrhythmias.^48^ These findings offer a novel mechanistic link between Cx43 hemichannel dysfunction and the fatal sudden cardiac death events frequently observed in DMD patients.^78,79^

Along these lines, multiple reports have documented distinctive signs of myocardial injury in DMD patients,^4,5,80^ such as pathological QRS complexes,^81,82^ elevated cTnI levels in the blood,^6,26,83^ and elevated ST segments.^74,82^ However, the aetiological mechanisms underlying these clinical manifestations remain unclear. Importantly, Cx43 remodelling has been implicated in myocardial infarction and arrhythmia susceptibility.^84^ Recent studies have identified four missense variants in GJA1, the gene encoding Cx43, in patients experiencing severe arrhythmias,^84^ which support the involvement of Cx43 in arrhythmogenicity. In this study, for the first time, we explored the role of cardiac Cx43 hemichannels in myocardial injury within the context of DMD cardiomyopathies. Our data reveal that 4 h post-isoproterenol challenge, DMD^mdx^ mice exhibit elevated plasma concentrations of cTnI, mirroring the increases reported in DMD patients and mouse models following myocardial injury.^11^ Remarkably, this elevation was abolished in DMD^mdx^ previously treated with the Cx43 hemichannel blocker Gap19 and in DMD^mdx^:C271S^+/−^ mice, strongly suggesting that S-nitrosylation and activation of Cx43 hemichannels mediate myocardial injury. Additionally, our findings indicate that 24 h post-isoproterenol stimulation; DMD^mdx^ mice display significantly larger infarct areas. However, the infarct size was effectively reduced in DMD^mdx^ mice treated with Gap19 and in DMD^mdx^:C271S^+/−^ mice. These results position Cx43 hemichannel dysfunction as a critical and previously unrecognized driver of myocardial injury and bradyarrhythmia in DMD cardiomyopathy. While mitochondrial Cx43 has also been implicated in cardiac injury (e.g.ischaemia–reperfusion),^85–87^ and may contribute to myocardial damage in this model, it is unlikely to account for the acute electrical instability observed here, which is more directly attributable to sarcolemma hemichannel activity. Overall, our findings establish a mechanistic link between Cx43 hemichannel activation and myocardial injury in DMD and highlight these channels as a potential therapeutic target to reduce arrhythmias and cardiac damage.

In conclusion, our findings reveal that cardiac stress–induced S-nitrosylation of Cx43 at cysteine 271 is a key mechanistic driver of lateralized Cx43 hemichannel hyperactivity in the dystrophic ventricle. This post-translational modification disrupts Ca^2+^ signalling, prolongs APs, promotes TA and conduction block, leading to fatal arrhythmias, bradycardia, and myocardial injury in DMD mice. These results highlight the critical role of Cx43 S-nitrosylation at Cys271 in arrhythmogenesis and myocardial damage, identifying them as promising therapeutic targets to prevent lethal cardiac complications in DMD.

### 4.1 Study limitations

The DMD^mdx^ mouse model has been invaluable for elucidating the mechanisms underlying DMD-associated cardiomyopathy and arrhythmogenesis, particularly through intracellular channel dysregulation. However, its translational relevance is limited by the model’s milder cardiac phenotype and longer lifespan compared with human DMD patients, who typically develop early-onset dilated cardiomyopathy.^52,88^ Optical mapping using the voltage-sensitive dye RH-237 is a powerful tool for assessing membrane voltage and APD, offering high spatial resolution and effective dual *V_m_*-Ca^2+^ imaging in various species.^89–93^ Nevertheless, in DMD^mdx^ hearts, the RH-237 signal amplitude is notably reduced. This attenuation may stem from early membrane susceptibility common in DMD hearts, which disrupts uniform dye distribution and increases photon scattering, possibly linked to altered membrane density.^94,95^ Despite these limitations, the AP properties observed in DMD^mdx^ hearts via optical mapping are consistent with those measured in isolated cardiomyocytes using patch-clamp recordings,^19^ supporting the relevance of the optical mapping data.

## Supplementary Material

cvag170_Supplementary_Data

## Data Availability

The data supporting the findings of this study are available from the corresponding author upon reasonable request.
