# Supplementary material for: Reduced Cx43 S-nitrosylation protects against cardiac stress–induced arrhythmias and myocardial injury in Duchenne muscular dystrophy
Source: Cardiovasc Res. 2026 Aug 10;122(14):1947–63. doi: 10.1093/cvr/cvag170 (PMC13653503; doi:10.1093/cvr/cvag170)
Supplement: Supplementary file 1 [file cvag170_supplementary_data.docx]

**Reduced Cx43 S-nitrosylation protects against cardiac stress–induced arrhythmias and myocardial injury in Duchenne Muscular Dystrophy**

Manuel F. Muñoz^1^, Ph.D., Pablo S. Gaete^1^, Ph.D., Jonathan J. Quan^1^, B.Sc., Thao T. Nguyen^1^, B.Sc., Janet Nuno^1^, B.Sc., Adrian Sheehy^1^, Priscila A. Araujo^1^, Ph.D., Pia C. Burboa^2^, Ph.D., Mauricio A. Lillo^2^, Ph.D., Jorge E. Contreras^1‡^, Ph.D.

**Affiliations:**

1. Department of Physiology and Membrane Biology, University of California, Davis.

2. Department of Pharmacology, Physiology and Neuroscience, Rutgers University, New Jersey Medical School.

**Corresponding Author:**

^‡^Dr. Jorge E. Contreras; University of California, Davis, School of Medicine, 4144 Tupper Hall; One Shields Avenue, CA 95616. [jecontrer@ucdavis.edu](mailto:jecontrer@ucdavis.edu)

**SUPPLEMENTAL MATERIAL**

**Online Figure S1-S11**

**Supplementary Figure 1. Nitric oxide (NO) donors induce hemichannel opening in oocytes expressing Cx43 hemichannels.** (A) Quantification of the current-voltage relationship in non-injected oocytes (NI) (black circles), oocytes expressing wild-type Cx43 (Cx43^WT^) (white circles), the C271S mutant (Cx43^C271S^) (white rhombus), and wild-type Cx43 (Cx43^WT^): C271S mutant (Cx43^WT/C271S^), representing a heterozygous C271S mutant (white triangle), under control conditions (left panel) and in the presence of 100 µM diethylamine NONOate (DEA NONOate) (right panel). (B) Quantification of currents at -70 mV (left panel) and + 70 mV (right panel) in oocytes under control conditions (black circles) and in the presence of 100 µM DEA NONOate (red circles). Each dot represents an independent oocyte. Group comparisons were made using a two-way ANOVA followed by Tukey’s post hoc test. Data are presented as means ± SEM.

**Supplementary Figure 2. Characterization of Cx43^C271S^ mutants.** (A) Schematic representation of the knock-in mouse model Cx43 developed using CRISPR-Cas9 genome editing, in which the site of S-nitrosylation of Cx43, cysteine 271 (C271), was replaced by a serine (C271S). (B) Representative western blots showing levels of S-nitrosylated Cx43 proteins (Cx43 S-NO) in heterozygous Cx43:C271S^+/-^ (C271S^+/-^) and homozygous Cx43:C271S^+/+^ (C271S^+/+^) mouse hearts. The lower blot is the Ponceau staining of the total pull-down for S-nitrosylated proteins. Each lane represents an individual mouse heart. (C) Quantification of body weight, heart weight (D), and heart weight/body weight ratio (E) from wild-type (black), C271S^+/-^ (green), and C271S^+/+^ (blue) mice. (F) Quantification of mean arterial pressure (MAP) measured by a telemetry system in the carotid artery of mice. Each dot represents an independent animal. Group comparisons were made using a one-way ANOVA followed by Tukey’s post hoc test. Data are presented as means ± SEM.

**Supplementary Figure 3. DMD^mdx^:C271S^+/-^ knockin mice exhibit reduced S-nitrosylation of cardiac Cx43 proteins.** (A) The top membrane was loaded with S-nitrosylated proteins isolated from heart samples from wild-type (WT), DMD^mdx^, and DMD^mdx^:C271S^+/-^ mice using the biotin switch assay. This membrane was probed for connexin 43 (Cx43). The middle panel shows the total cardiac proteins blotted for Cx43, and the bottom panel shows GAPDH used as a loading control. (B) Quantification of S-nitrosylated Cx43 (Cx43-SNO), shown as fold difference relative to WT. Each dot represents an independent animal. Group comparisons were made using a one-way ANOVA followed by Tukey’s post hoc test. Data are presented as means ± SEM.

**Supplementary Figure 4. Cardiac hypertrophy observed in DMD^mdx^ is prevented in DMD^mdx^:C271S^+/^ mice.** (A) Quantification of body weight, heart weight (B), and heart weight/body weight ratio (C) from wild-type (white circles), DMD^mdx^ (blue circles), DMD^mdx^:C271S^+/-^ (orange circles), and C271S^+/-^ (green circles) mice (4- to 6-month-old mice). Each dot represents an independent animal. Group comparisons were made using a one-way ANOVA followed by Tukey’s post hoc test. Data are presented as means ± SEM.

**Supplementary Figure 5. Decreased levels of S-nitrosylated Cx43 hemichannels in DMD^mdx^ mice prevent the prolongation of action potential durations (APDs) observed in DMD^mdx^ mice after β-adrenergic overstimulation.** (A) Representative heat maps of action potential duration at 90% repolarization (APD_90_) of wild-type, DMD^mdx^, and DMD^mdx^:C271S^+/-^ hearts before (baseline) and during adrenergic overstimulation with isoproterenol treatment (4 µM). (B) Representative optical mapping traces of APDs at baseline conditions and following exacerbated β-adrenergic stimulation (isoproterenol) in wild-type (black), DMD^mdx^ (red), and DMD^mdx^:C271S^+/-^ (orange) hearts previously loaded with the voltage-sensitive probe RH237 via Langendorff perfusion system. (C) Action potential durations: APD_90_, APD_50_, APD_30_ before (white circles) and after isoproterenol stimulation (blue circles). APDs were calculated by the average of 6 independent regions of interest in the ventricular area of the mouse heart. *n*=5 mice per group. Each dot represents an independent animal. Group comparisons were made using a two-way ANOVA followed by Tukey’s post hoc test. Data are presented as means ± SEM.

**Supplementary Figure 6. S-nitrosylation of Cx43 channels in DMD^mdx^ mice mediates increased cardiac Ca^2+^ signaling after β-adrenergic overstimulation.** (A) Representative heat maps of Ca^2+^ amplitudes of wild-type, DMD^mdx^, and DMD^mdx^:C271S^+/-^ hearts before (baseline) and during exacerbated β-adrenergic stimulation with isoproterenol treatment (4 µM). (B) Representative Ca^2+^ traces at baseline conditions and following β-adrenergic stimulation (4 µM, isoproterenol) in wild-type (black), DMD^mdx^ (red), and DMD^mdx^:C271S^+/-^ (orange) in hearts previously loaded with the Ca^2+^ indicator Rhod-2 via Langendorff perfusion system. (C) Quantification of Ca^2+^ amplitude (left panel) and decay constant (right panel) ratio before and post-overstimulation of β-adrenergic receptors with 4 µM isoproterenol. Ca^2+^ amplitudes were calculated as the average of 6 independent regions of interest in the ventricular area of the mouse heart. *n*=5-6 mice per group. Each dot represents an independent animal. Group comparisons were made using a one-way ANOVA followed by Tukey’s post hoc test. Data are presented as means ± SEM.

**Supplementary Figure 7. S-nitrosylation of cardiac Cx43 hemichannels in DMD^mdx^ mice mediates conduction block, enhances Ca^2+^ signaling, and increases diastolic interval heterogeneity in response to frequency stimulation and β-adrenergic receptor overstimulation.** (A) Representative optical mapping traces of action potentials (AP, top traces, black) and Ca^2+^ signals (bottom traces, blue) in wild-type (black), DMD^mdx^ (red), and DMD^mdx^:C271S^+/-^ (orange) hearts at baseline (left), during (middle), and 2 minutes (right) after 10 Hz burst pacing for 1 minute + 4 μM of isoproterenol (Iso) (10 Hz + Iso) stimulation. Scale bar: 0.5 s. (B) Average coefficient of variation (COV, as a percentage) of diastolic intervals between cardiac action potentials at baseline, 10 Hz + Iso, and 2 minutes after electrical stimulation in wild-type (white circles), DMD^mdx^ (red circles), and DMD^mdx^:C271S^+/-^ (orange circles) hearts. The diastolic intervals were obtained from diastolic time intervals among APs, calculated as the average of 6 consecutive beats in 6 independent regions of interest in the ventricular area of the mouse heart. Group comparisons were made using two-way ANOVA followed by Tukey’s post hoc test. *n*=5-7 mice per group. Each dot represents an independent animal.

**Supplementary Figure 8. DMD^mdx^:C271S^+/−^ cardiomyocytes are protected against isoproterenol-induced triggered activity.** (A) Representative action potential recordings from DMD^mdx^ and DMD^mdx^:C271S^+/−^ cardiomyocytes under control conditions and after isoproterenol stimulation. Scale bars: 50 mV and 250 ms. (B) Quantification of triggered activity observed during 1 minute in DMD^mdx^, DMD^mdx^ + Iso, DMD^mdx^:C271S^+/−^, and DMD^mdx^:C271S^+/−^ + Iso cardiomyocytes. Each dot represents an independent cell. Group comparisons were made using a one-way ANOVA followed by Tukey’s post hoc test. *n*=3-5 mice per group. Data are presented as means ± SEM.

**Supplementary Figure 9. Wild-type, DMD^mdx^, and DMD^mdx^: C271S^+/−^ cardiomyocytes display similar L-type Ca^2+^ currents.** (A) Representative normalized I_Ca_ traces recorded from wild-type, DMD^mdx^, and DMD^mdx^: C271S^+/−^ cardiomyocytes. Scale bar: 5 ms. (B) Quantification of I_Ca_ density (pA/pF) in wild-type, DMD^mdx^, and DMD^mdx^:C271S^+/−^ cardiomyocytes. Data are presented as mean ± SEM. Each circle represents an independent cardiomyocyte. *n* = 3-9 mice per group. Group comparisons were made using a one-way ANOVA followed by Tukey’s post hoc test.

**Supplementary Figure 10. Uncropped and unedited blots used in Supplementary Figure 2**. The dotted red line indicates the region corresponding to the molecular weight of S-nitrosylated Cx43 (A) and the Ponceau staining of the total pull-down of S-nitrosylated proteins (B).

**Supplementary Figure 11. Uncropped and unedited blots used in Supplementary Figure 3**. The dotted red line indicates the region corresponding to the molecular weight for S-nitrosylated Cx43 (A), total Cx43 (B), and GAPDH (C).
